# Identification and allele mining of new candidate genes underlying rice grain weight and grain shape by genome-wide association study

**DOI:** 10.1186/s12864-021-07901-x

**Published:** 2021-08-06

**Authors:** Yanan Niu, Tianxiao Chen, Chunchao Wang, Kai Chen, Congcong Shen, Huizhen Chen, Shuangbing Zhu, Zhichao Wu, Tianqing Zheng, Fan Zhang, Jianlong Xu

**Affiliations:** 1 Institute of Crop Sciences, National Key Facility for Crop Gene Resources and Genetic Improvement, Chinese Academy of Agricultural Sciences, 100081 Beijing, China; 2 Tasmanian Institute of Agriculture, University of Tasmania, 7250 Prospect, TAS Australia; 3 Guangdong Laboratory of Lingnan Modern Agriculture, Genome Analysis Laboratory of the Ministry of Agriculture, Agricultural Genomics Institute at Shenzhen, Chinese Academy of Agricultural Sciences, Shenzhen, China; 4 Pingxiang Institute of Agricultural Sciences, 337000 Pingxiang, China

**Keywords:** Rice germplasm, Grain shape, Grain weight, Quantitative trait nucleotides (QTNs), Favorable haplotype

## Abstract

**Background:**

Grain weight and grain shape are important agronomic traits that affect the grain yield potential and grain quality of rice. Both grain weight and grain shape are controlled by multiple genes. The 3,000 Rice Genomes Project (3 K RGP) greatly facilitates the discovery of agriculturally important genetic variants and germplasm resources for grain weight and grain shape.

**Results:**

Abundant natural variations and distinct phenotic differentiation among the subgroups in grain weight and grain shape were observed in a large population of 2,453 accessions from the 3 K RGP. A total of 21 stable quantitative trait nucleotides (QTNs) for the four traits were consistently identified in at least two of 3-year trials by genome-wide association study (GWAS), including six new QTNs (*qTGW3.1*, *qTGW9, qTGW11*, *qGL4/qRLW4*, *qGL10*, and *qRLW1*) for grain weight and grain shape. We further predicted seven candidate genes (*Os03g0186600*, *Os09g0544400*, *Os11g0163600*, *Os04g0580700*, *Os10g0399700*, *Os10g0400100* and *Os01g0171000*) for the six new QTNs by high-density association and gene-based haplotype analyses. The favorable haplotypes of the seven candidate genes and five previously cloned genes in elite accessions with high TGW and RLW are also provided.

**Conclusions:**

Our results deepen the understanding of the genetic basis of grain weight and grain shape in rice and provide valuable information for improving rice grain yield and grain quality through molecular breeding.

**Supplementary Information:**

The online version contains supplementary material available at 10.1186/s12864-021-07901-x.

## Background

Rice (*Oryza sativa* L.) is one of the most widely grown crops in the world and is a staple diet for more than 3.5 billion people around the world, particularly in Asia. It is predicted that rice production must increase by ∼42 % by 2050 to keep pace with increasing global food demand [1]. As the main component of grain yield in rice, grain weight or thousand-grain weight (TGW) has always been a dominating trait for breeders. Meanwhile, grain shape is an important factor affecting physical or appearance quality, where slender grains with grain length-to-width ratio > 3 are preferred by most rice consumers [2]. Grain shape is determined by grain length (GL), grain width (GW), the ratio of length-to-width (RLW) and grain thickness, and grain weight is positively correlated with these four traits [3].

Over the past few decades, more than 60 genes associated with grain weight and grain shape have been identified in rice [4]. To date, more than 10 major quantitative trait loci (QTL) controlling grain weight and grain shape have been cloned and characterized [5–9]. Among them, *GW2*, *GW7*/*GL7*, *GW8*/*OsSPL16* and *GS9* regulate grain shape through altering cell division with influences on appearance quality like chalkiness [7, 10–13]. Moreover, several QTL for grain weight or grain shape have been identified, such as *qGRL7.1* [14], *qGL4b* [15], *qSS7* [16] and *tgw11* [17], but the causal genes are yet to be characterized.

Next-generation sequencing (NGS) technologies have made genotyping more efficient and the availability of substantially increased SNP markers enables the exploration of quantitative trait nucleotides (QTNs)/genes for target traits more rapidly through genome-wide association study (GWAS), which has been widely applied in genetic dissection of agronomic traits in rice [18–22]. So far, several genes influencing grain weight and grain shape have already been identified by GWAS in rice, such as *GLW7* for GL and TGW [23], *OsLG3* for TGW and GL [24], *GSE5* regulating grain width [25], and *OsSNB* controlling grain size [26]. Moreover, GWAS is also a powerful method to explore favorable alleles associated with important agronomic traits in rice natural populations and germplasm [27, 28]. Recently, the 3,000 Rice Genomes Project (3 K RGP) contributed 29 million single nucleotide polymorphisms (SNPs), 2.4 million small InDels and over 90,000 structural variations [29, 30]. The availability of this genomic data provides a valuable genetic resource for both scientific research and molecular breeding in rice [31, 32].

Here, we conducted a GWAS with a high-density SNP dataset using 2,453 accessions from the 3 K RGP followed by gene-based haplotype analysis to identify new candidate genes underlying rice grain weight and grain shape. The results of this study will enhance the understanding of the genetic basis of grain weight and grain shape, and provide valuable information for improving grain shape and grain yield in rice breeding.

## Results

### Phenotypic variations and correlations

**Fig. 1:**
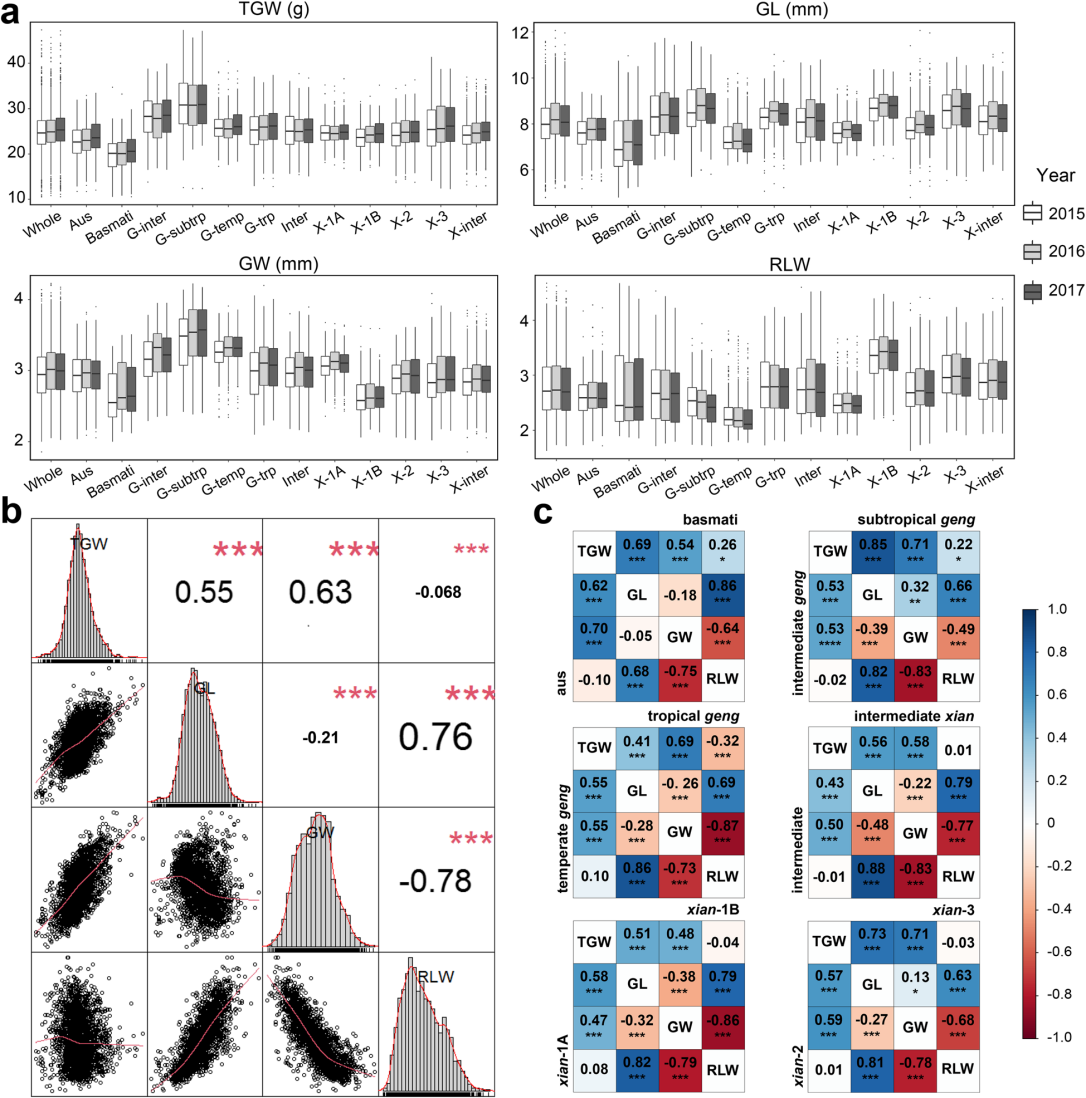
Phenotypic distributions and correlations. **a** Box plots of TGW, GL, GW, and RLW in three years across 12 subgroups. TGW: thousand grain weight; GL: grain length; GW: grain width; RLW: ratio of GL to GW; G-inter: intermediate *geng*; G-subtrp: subtropical *geng*; G-tem: temperate *geng*; Inter: intermediate; X-1 A: *xian*-1 A; X-1B: *xian*-1B; X-2: *xian*-2; X-3: *xian*-3; X-inter: intermediate *xian*. **b** Distributions and correlations among the four traits in the whole population. **c** Correlations among the four traits from each subgroup. The number in the middle of the cell is the correlation coefficient; ‘*’, ‘**’, and ‘***’ refer to significant correlations (*P* < 0.05, *P* < 0.01, and *P* < 0.001)

All four measured traits showed large variations in the whole population and between subgroups across the three years (Fig. 1a, Additional file 1: Table S2 and Table S3). ‘Nyao’ from Laos had the highest TGW (46.60 g), ‘GEANT W 7’ from Netherlands had the longest GL (11.82 mm), ‘DO LEUANG’ from Laos had the widest GW (4.17 mm), and ‘IET 14720’ had the largest RLW (4.55) (Additional file 1: Table S3). Among the 12 subgroups, subtropical *geng* had the highest mean TGW (31.38 g) primarily due to significantly longer GL (8.68 mm) and wider GW (3.51 mm) compared to the other subgroups, followed by intermediate *geng* and *xian*-3 (Additional file 1: Table S3). Moreover, *xian*-1B had the highest mean GL (8.73 mm) and RLW (3.34) while the overall lowest mean GW was found in the *xian*-1B subgroup. The *basmati* subgroup showed significantly lower TGW (19.86 g) and GL (7.25 mm) than all the other subgroups. Although the *xian*-1B, *xian*-3, and subtropical *geng* subgroups had similar GL, the two *xian* subgroups (*xian*-1B and *xian*-3) showed significantly larger RLW than subtropical *geng*.

TGW showed a significant positive correlation with both GL and GW in the whole population and 12 subgroups, with the strongest correlation (*r* = 0.85 and *r* = 0.71, *P* < 0.001) detected in the subtropical *geng* subgroup (Fig. 1b and c). GL was negatively correlated with GW in the whole population and most subgroups except for subtropical *geng* (*r* = 0.32, *P* < 0.01) and *xian*-3 (*r* = 0.13, *P* < 0.05). TGW was significantly negatively correlated with RLW in tropical *geng* subgroup (*r* = -0.32, *P* < 0.001) while a significant positive correlation between TGW and RLW was detected in the *basmati* (*r* = 0.26, *P* < 0.05) and subtropical *geng* (*r* = 0.22, *P* < 0.05) subgroups. All the four traits showed very high heritability ranging from 0.88 for GL to 0.93 for GW (Table 1).

**Table 1:** Variance components and heritability estimates for grain weight and grain shape-related traits

Trait	V_g_	V_E_	V_gei_	V_rep_	V_e_	*h*^*2*^
TGW	16.69	7.51	3.49	0.28	1.24	0.92
GL	0.84	0.13	0.32	0.03	0.05	0.88
GW	0.122	0.051	0.021	0.011	0.009	0.93
RLW	0.269	0.103	0.067	0.003	0.005	0.92

*V*_*g*_ genotype variance, *V*_*E*_ environment variance, V_*gei*_ genotype by environment interaction variance, *V*_*rep*_ replication variance within environment, *V*_*e*_ residual variance, *h*^*2*^ narrow-sense heritability. Trait abbreviations are as given in the legend to Fig. 1

### Genome-wide LD patterns and QTN detection by GWAS

The maximum LD was 0.47, 0.64, 0.69, 0.53, 0.68, and 0.67 in the whole population, *aus*, *basmati*, *xian*, temperate *geng* and tropical *geng*, respectively. LD reached half of its initial value at around 300 kb in the whole population, *basmati* and temperate *geng* subgroups, 250 kb in the *aus* subgroup, 130 kb in the *xian* group and 260 kb in the tropical *geng* subgroup (Additional file 2: Fig. S1). Thus, the LD decay in the *xian* subgroup was much faster than in any of the other subgroups.

A total of 21 stable QTNs for the four traits were consistently identified in at least two years (Table 2; Additional file 2: Fig. S2A–S4F). For TGW, six QTNs were detected on chromosomes 3, 5, 7, 9, and 11. Among them, *qTGW3.2* (rs3_16733441), *qTGW5* (rs5_5371529), and *qTGW7* (rs7_28305040) were stably detected in all three years with *qTGW7* showing the strongest association signal (*P* = 8.12E-18) in the whole population. Two QTNs, *qTGW3.1* (rs3_4504988) and *qTGW9* (rs9_21404841), were detected only in the whole population while *qTGW11* (rs11_3019935) was detected only in the *xian* subgroup.

**Table 2:** QTNs consistently identified for grain weight and grain shape in at least two years by GWAS

Trait ^a^	QTN ^b^	Chr.	Lead SNP ^c^	Allele ^d^	*P*-value	Population/Subgroup	Year of QTN detected	Known gene
TGW (g)	*qTGW3.1*	3	rs3_4504988	C/T	2.59E-08	Whole	2015, 2016	
	*qTGW3.2*	3	rs3_16733441	T/G	2.25E-08	Whole	2015–2017	*GS3* [33]
		3	rs3_16733441	T/G	1.78E-11	*Xian*	2015–2017	
	*qTGW5*	5	rs5_5371529	C/A	7.22E-10	Whole	2015–2017	*qSW5*/*GW5* [34]
		5	rs5_5361877	T/A	1.38E-10	*Xian*	2015–2017	
	*qTGW7*	7	rs7_28305040	A/G	8.12E-18	Whole	2015–2017	*FZP* [35]
		7	rs7_28362557	G/A	7.37E-08	*Xian*	2015–2017	
		7	rs7_28322588	T/C	1.04E-09	Basmati	2015–2017	
		7	rs7_28287280	A/G	9.14E-12	Aus	2015–2017	
	*qTGW9*	9	rs9_21404841	C/T	2.47E-08	Whole	2015, 2017	
	*qTGW11*	11	rs11_3019935	T/C	5.17E-07	*Xian*	2016, 2017	
GL (mm)	*qGL3*	3	rs3_16733441	T/G	1.97E-73	Whole	2015–2017	*GS3* [33]
		3	rs3_16733441	T/G	8.65E-59	*Xian*	2015–2017	
		3	rs3_16733441	T/G	2.54E-15	Temperate *geng*	2016, 2017	
		3	rs3_16733441	T/G	5.16E-09	Tropical *geng*	2015–2017	
		3	rs3_16728308	C/T	4.75E-09	Aus	2016, 2017	
	*qGL4*	4	rs4_29308534	C/T	1.79E-06	Whole	2015, 2017	
	*qGL5*	5	rs5_5373491	A/G	2.49E-21	Whole	2015–2017	*qSW5*/*GW5* [34]
		5	rs5_5361894	A/G	2.09E-18	*Xian*	2015–2017	
		5	rs5_5351264	T/A	3.44E-08	Tropical *geng*	2016, 2017	
		5	rs5_5288638	G/A	2.58E-07	Aus	2015–2017	
	*qGL7*	7	rs7_28289869	T/C	4.62E-19	Whole	2015–2017	*FZP* [35]
		7	rs7_28287400	C/G	7.12E-07	*Xian*	2016, 2017	
		7	rs7_28290297	G/A	1.40E-10	Tropical *geng*	2015–2017	
		7	rs7_28287280	A/G	7.82E-08	Basmati	2016, 2017	
	*qGL10*	10	rs10_13616240	A/C	1.19E-07	Whole	2015–2017	
GW (mm)	*qGW3*	3	rs3_16733441	T/G	6.82E-09	Whole	2015–2017	*GS3* [33]
		3	rs3_16717406	G/A	2.21E-08	*Xian*	2015–2017	
	*qGW5*	5	rs5_5371772	G/A	1.84E-77	Whole	2015–2017	*qSW5*/*GW5* [34]
		5	rs5_5361894	A/G	1.96E-71	*Xian*	2015–2017	
		5	rs5_5364791	T/C	3.73E-10	Temperate *geng*	2015–2017	
		5	rs5_5375786	G/A	2.07E-25	Tropical *geng*	2015–2017	
		5	rs5_5346606	T/A	2.16E-19	Aus	2015–2017	
	*qGW7*	7	rs7_24898274	A/T	2.59E-16	Whole	2015–2017	*GL7/GW7* [11, 12]
		7	rs7_24902815	A/G	3.17E-07	Tropical *geng*	2015–2017	
	*qGW8*	8	rs8_26504638	A/G	3.58E-10	Aus	2016, 2017	*GW8* [13]
RLW	*qRLW1*	1	rs1_3657795	A/G	1.42E-07	Aus	2015, 2017	
	*qRLW3*	3	rs3_16733441	T/G	2.50E-55	Whole	2015–2017	*GS3* [33]
		3	rs3_16733441	T/G	4.00E-43	*Xian*	2015–2017	
		3	rs3_16733441	T/G	4.11E-07	Temperate *geng*	2015–2017	
		3	rs3_16728485	G/C	6.65E-11	Tropical *geng*	2016, 2017	
		3	rs3_16686074	G/A	7.33E-08	Basmati	2015–2017	
	*qRLW4*	4	rs4_29317460	C/A	3.68E-09	Whole	2015–2017	
		4	rs4_29309086	C/G	1.14E-08	Aus	2015–2017	
	*qRLW5*	5	rs5_5371609	A/G	3.59E-69	Whole	2015–2017	*qSW5*/*GW5* [34]
		5	rs5_5361894	A/G	1.25E-67	*Xian*	2015–2017	
		5	rs5_5364791	T/C	9.49E-12	Temperate *geng*	2015–2017	
		5	rs5_5375793	G/A	5.48E-25	Tropical *geng*	2016, 2017	
		5	rs5_5375764	A/G	5.56E-15	Aus	2015–2017	
	*qRLW7*	7	rs7_24533303	T/C	4.89E-13	Whole	2015–2017	*GL7*/*GW7* [11, 12]
		7	rs7_24537476	C/T	3.67E-07	*Xian*	2015–2017	
		7	rs7_24902815	A/G	8.02E-07	Tropical *geng*	2016, 2017	
	*qRLW8*	8	rs8_26504638	A/G	1.17E-14	Aus	2015–2017	*GW8* [13]

^a^Trait abbreviations are as given in the legend to Fig. 1

^b^A QTN was considered as a local LD block region where the significant trait-associated SNPs were located

^c^The SNP with the minimum *P*-value

^d^Major/Minor allele

Five QTNs for GL were mapped to chromosomes 3, 4, 5, 7, and 10. Three QTNs, *qGL3* (rs3_16733441), *qGL5* (rs5_5373491), and *qGL7* (rs7_28289869) were consistently detected in the whole population and subgroups with *qGL3* showing the strongest association signal (*P* = 1.97E-73) in the whole population. The QTNs, *qGL4* (rs4_29308534) and *qGL10* (rs10_13616240), were detected only in the whole population.

Four QTNs for GW were detected on chromosomes 3, 5, 7, and 8. Three QTNs, *qGW3* (rs3_16733441), *qGW5* (rs5_5371772), and *qGW7* (rs7_24898274) were stably detected in all three years with *qGW5* showing the strongest association signal (*P* = 1.84E-77) in the whole population. One QTN, *qGW8* (rs8_26504638) was detected only in the *aus* subgroup in 2016 and 2017.

Six QTNs for RLW were identified on chromosomes 1, 3, 4, 5, 7, and 8. Four QTNs, *qRLW3* (rs3_16733441), *qRLW4* (rs4_29317460), *qRLW5* (rs5_5371609), and *qRLW7* (rs7_24533303) were consistently detected in the whole population and subgroups with *qRLW5* showing the strongest association signal (*P* = 3.59E-69) in the whole population. Two QTNs, *qRLW1* (rs1_3657795) and *qRLW8* (rs8_26504638) were detected only in the *aus* subgroup.

We specified QTNs with overlapping regions identified for multiple traits as the same QTN, which lead to the identification of 11 different QTNs. Among them, *qTGW3.2*/*qGL3*/*qGW3*/*qRLW3*, *qTGW5*/*qGL5*/*qGW5*/*qRLW5*, *qTGW7*/*qGL7*, *qGW7/qRLW7*, and *qGW8*/*qRLW8* were actually *GS3*, *qSW5*/*GW5*, *FZP*, *GL7*/*GW7*, and *GW8*, respectively, which are well-known genes controlling rice grain shape and grain weight. Notably, *qTGW3.1*, *qTGW9*, *qTGW11*, *qGL4/qRLW4*, *qGL10*, and *qRLW1* were newly identified in this study (Table 2).

### Candidate genes identification and haplotype analyses

**Fig. 2:**
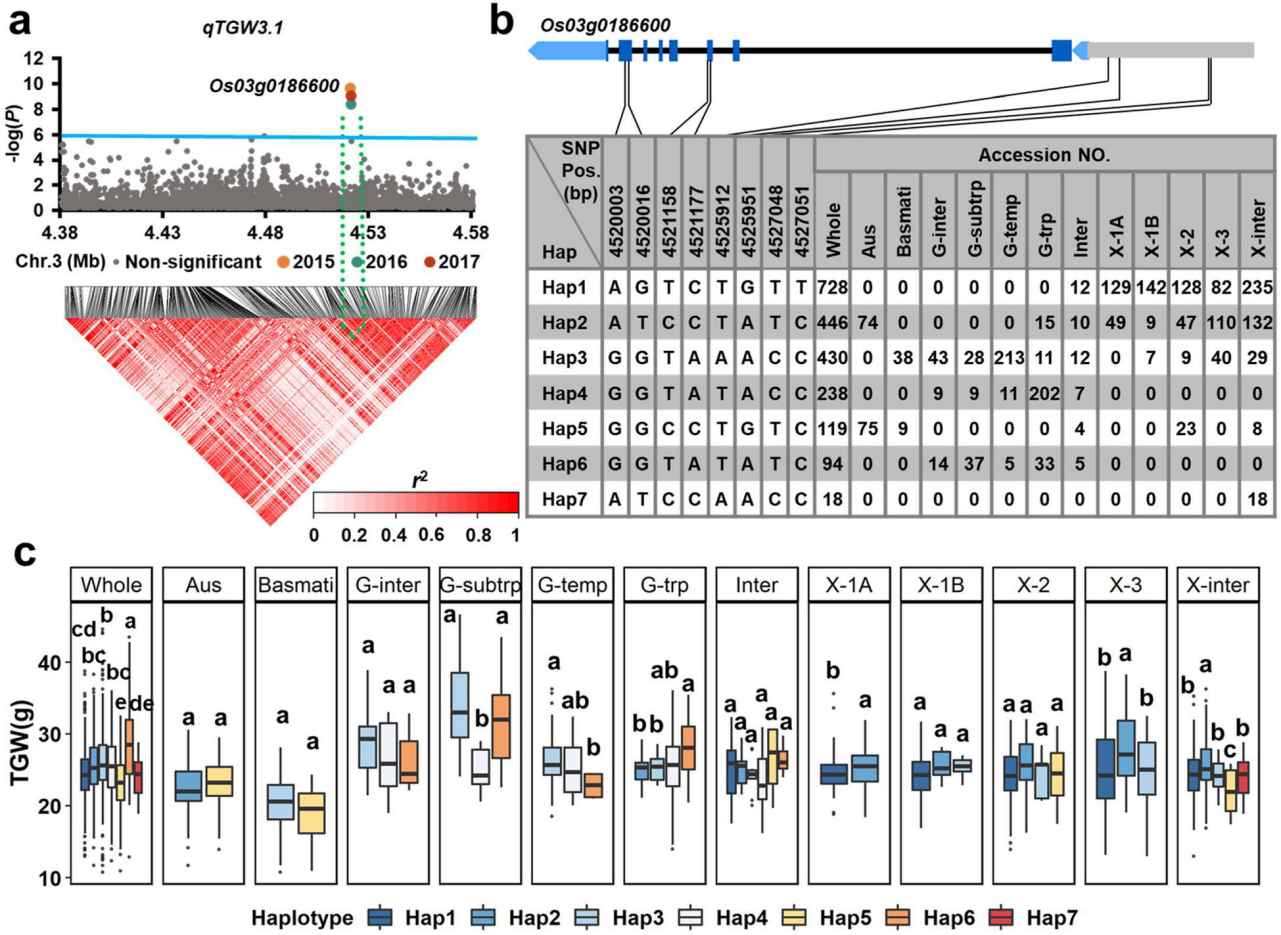
**a** High-density association analysis of *qTGW3.1* in 2015, 2016, and 2017. The solid line indicates the threshold to determine significant SNP. **b-c** Structure of candidate gene *Os03g0186600* and haplotype analysis for TGW in the whole population and the 12 subgroups. Characters on top of boxplots indicate significant differences based on Duncan’s multiple comparison tests (*P* < 0.05)

The six newly identified QTNs were used for the high-density association and gene-based haplotype analyses to identify the candidate genes. In the region of *qTGW3.1* (4.38–4.58 Mb on chromosome 3), 9,177 SNPs were used for high-density association analysis in the whole population. The annotated gene with the most significant hit was *Os03g0186600* (Fig. 2a). Seven major haplotypes were detected among 2,066 accessions based on four SNPs in the 2-kb region upstream of the *Os03g0186600* promoter, and four SNPs in the coding region (Fig. 2b). Significant differences for TGW were observed among the seven haplotypes in the whole population and several subgroups (Fig. 2c; Additional file 1: Table S4 and Table S5). In the whole population, Hap6, represented by 93 *geng* and intermediate accessions, had the highest mean TGW (28.68 g). Of the 73 subtropical *geng* accessions, 28 accessions with Hap3 had a mean TGW of 34.17 g and while a further 37 accessions had a mean TGW of 31.66 g. Hap2 showed a significantly higher TGW than the other haplotypes in the *xian*-1 A, *xian*-3, and intermediate *xian* subgroups (Fig. 2c).

The QTN *qTGW9* was identified in a 21.24–21.55 Mb region on chromosome 9, including 9,593 SNPs used for high-density association analysis in the whole population. The most significant hit was located in *Os09g0544400* (Additional file 2: Fig. S5). Three major haplotypes for *Os09g0544400* were detected among the 2,270 accessions. Notably, Hap2 and Hap3 were enriched in the *geng* and *xian* subgroups, respectively, suggesting that this gene differentiated between the two subspecies. Among them, Hap2 was associated with the highest TGW of 25.76 g in the whole population (Additional file 1: Table S4 and Table S5).

*qTGW11* was detected in the region from 2.82 Mb to 3.13 Mb on chromosome 11 in the *xian* subgroup, harboring 9,948 SNPs. *Os11g0163600* was subsequently screened as the candidate gene for *qTGW11* (Additional file 2: Fig. S6). A total of five haplotypes were identified in 2,056 accessions based on three SNPs in the 2-kb region upstream of the *Os11g0163600* promoter, one SNP in the 5’ UTR, one SNP in the coding region, and one SNP in the 3’ UTR. Only two haplotypes, Hap2 and Hap3, were detected in the four *geng* subgroups while Hap2 showed a significantly higher mean TGW than Hap3 in intermediate *geng* and tropical *geng* subgroups (Additional file 1: Table S4 and Table S5).

**Fig. 3:**
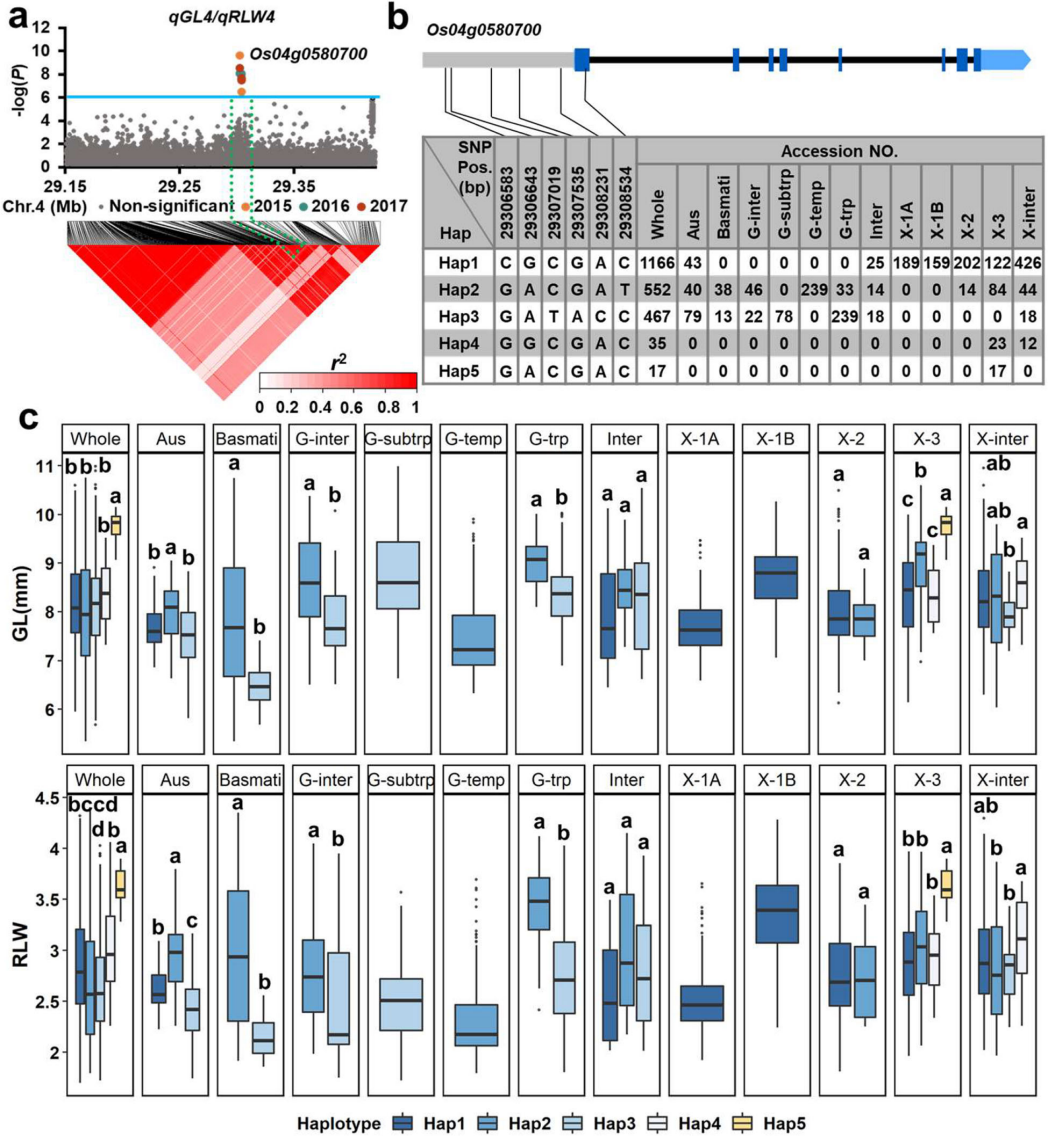
**a** High-density association analysis of *qGL4*/*qRLW4* in 2015, 2016, and 2017. The solid line indicates the threshold to determine significant SNP. **b-c** Structure of candidate gene *Os04g0580700* and haplotype analysis for GL and RLW in the whole population and the 12 subgroups. Characters on top of boxplots indicate significant differences based on Duncan’s multiple comparison tests (*P* < 0.05)

For *qGL4*/*qRLW4*, *Os04g0580700* was identified as the candidate gene (Fig. 3a). Five haplotypes were detected based on five SNPs in the 2-kb region upstream of its promoter and one SNP in the coding region (Fig. 3b). Hap5 was exclusively carried by 15 *xian*-3 accessions having the highest mean GL (9.72 mm) and mean RLW (3.62) (Fig. 3c). Moreover, Hap2 had a significantly higher GL and RLW than the other Haps in the *aus*, *basmati*, intermediate *geng*, and tropical *geng* subgroups (Fig. 3c; Additional file 1: Table S4 and Table S5).

Two candidate genes, *Os10g0399700* and *Os10g0400100*, were identified for *qGL10* (Additional file 2, Fig. S7). Four and six SNPs were used to classify haplotypes for *Os10g0399700* and *Os10g0400100*, resulting in five and six haplotypes, respectively (Additional file 2, Fig. S7). Interestingly, Hap4 of *Os10g0399700* and Hap5 of *Os10g0400100* both had a significantly higher mean GL than the other haplotypes in the whole population, which were only carried by the accessions of the four *geng* subgroups (Additional file 1: Table S4 and Table S5).

**Fig. 4:**
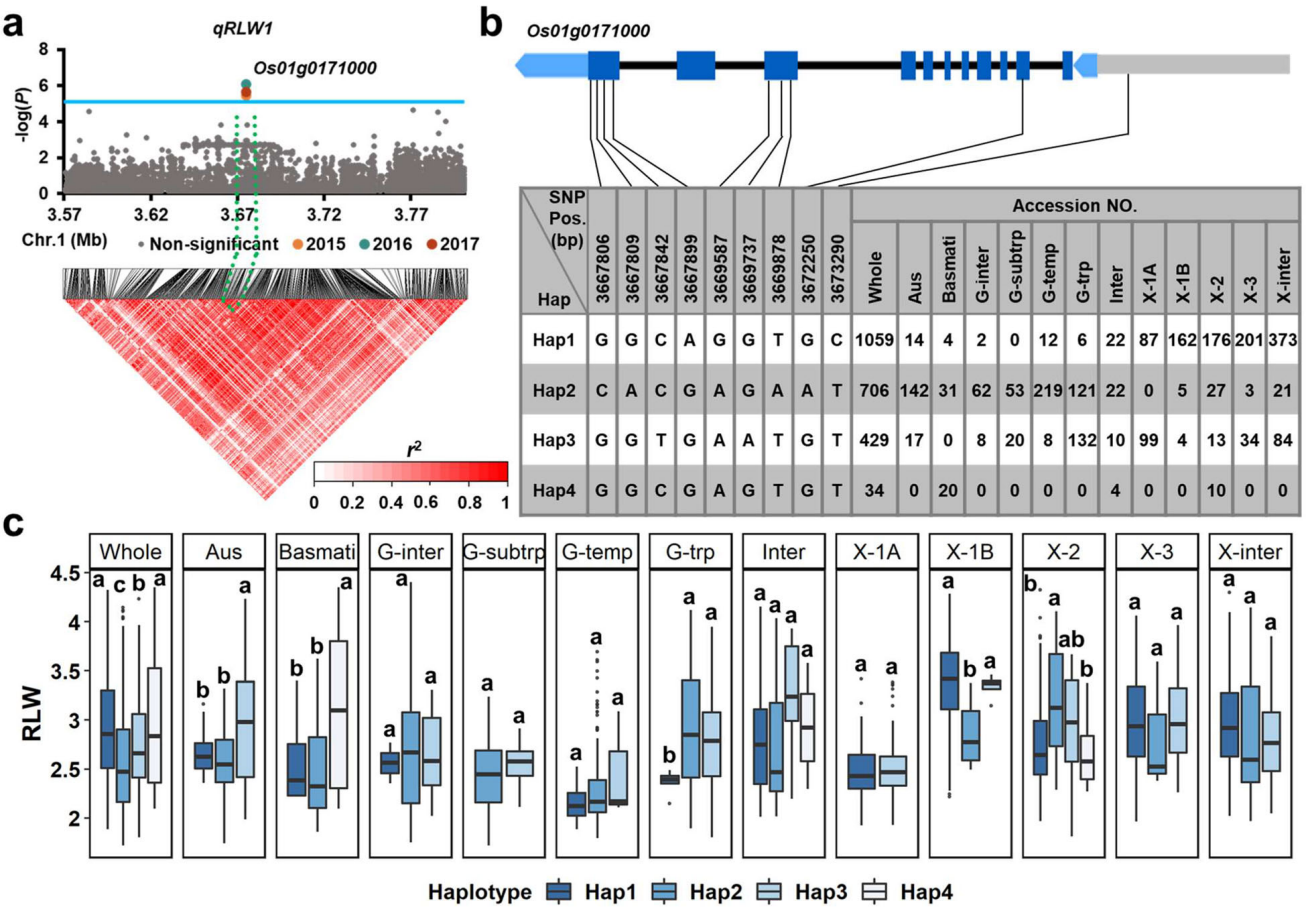
**a** High-density association analysis of *qRLW1* in 2015, 2016, and 2017. The solid line indicates the threshold to determine significant SNP. **b-c** Structure of candidate gene *Os01g0171000* and haplotype analysis for RLW in the whole population and the 12 subgroups. Characters on top of boxplots indicate significant differences based on Duncan’s multiple comparison tests (*P* < 0.05)

*qRLW1* was detected as an association peak of the region 3.57–3.80 Mb on chromosome 1 in the *aus* subgroup, including 8,116 SNPs used for high-density association analysis. The candidate gene with the most significant hit within an LD block was *Os01g0171000* (Fig. 4a). Four major haplotypes were observed in 2,224 accessions based on nine SNPs located in the 2-kb promoter and coding region of *Os01g0171000*. Of the four major haplotypes, Hap1 had a significantly higher mean RLW than that of Hap2 in the whole population. More notably, Hap1 was mainly carried by *xian* accessions while Hap2 was mainly present in the *geng* and *aus* accessions (Fig. 4c; Additional file 1: Table S4 and Table S5).

## Discussion

### Abundant variations of grain weight and grain shape in rice germplasm

Generally speaking, TGW, GL, and RLW vary greatly among rice varieties, while GW and grain thickness changed much less [5, 36–38]. In this study, the coefficient of variations of TGW, GL, GW, and RLW ranged from 10.38 to 22.55 %, 7.56 % to 18.61, 6.73–13.84 %, and 12.29–26.16 % in the 12 rice subgroups, respectively (Additional file 1: Table S2). The *basmati* subgroup showed the highest level of phenotypic variation for both grain weight and grain shape, indicating its genetic diversity can be further exploited for rice breeding [39, 40]. Two *xian* subgroups, *Xian*-1 A and *Xian*-1B, showed less phenotypic variation for grain weight and grain shape than the other subgroups. For TGW, *xian*-1B showed a mean value of 24.22 g that is close to 24.89 g in *xian*-1 A. For grain shape, *xian*-1B showed significantly higher GL (8.73 mm versus 7.71 mm) and RLW (3.34 versus 2.51), and significantly smaller GW (2.65 mm versus 3.10 mm) than *xian*-1 A (Additional file 1: Table S3). Interestingly, *xian*-1 A mainly consists of landraces and is closely related phylogenetically to *xian*-1B that largely consists of modern varieties [29]. These results suggest that many genes related to grain shape have also been strongly selected for in *xian*-1B, which is equivalent to the *IndII* group with various breeding signatures caused by geographic adaptation and accumulation of divergent selections in distinct breeding pools [41].

Although dominant effects have also been detected in some studies, most genes reported are predominantly additive in effect for TGW, GL, GW, and RLW [3]. Compared with other grain shape traits, grain length is the major determinant of grain weight [42]. In the current study, we found six stable QTNs that had pleiotropic effects on grain shape and grain weight. For instance, two QTNs (*qTGW3.2*/*qGL3*/*qGW3*/*qRLW3* and *qTGW5*/*qGL5*/*qGW5*/*qRLW5*) had pleiotropic effects on all four traits, and *qTGW7*/*qGL7* showed pleiotropic effects on grain weight and grain length. The power to obtain different combinations of alleles conferring particular grain shapes and sizes from almost every subgroup has implications for the improvement of yield and grain quality, potentially enabling breeders to develop high-yielding varieties with specific grain quality to satisfy diverse quality requirements.

### Identification of the favorable alleles among the previously cloned genes

Comparisons of QTNs detected in this study with the known genes for grain weight or grain shape were performed within 1 Mb physical distance around the lead SNP of each QTN based on the Nipponbare reference genome IRGSP-1.0. Of the 11 QTNs, five were found to co-locate in the same regions as previously cloned genes related to grain weight or grain shape in rice (Table 2). *qTGW3.2*/*qGL3*/*qGW3*/*qRLW3* was mapped close to *GS3*, a major gene for grain size previously identified in the *xian* varieties [33]. *qTGW5*/*qGL5*/*qGW5*/*qRLW5* covered *qSW5/GW5* which regulates grain width and weight [34]. *qGW7*/*qLWR7* was co-located with *GL7/GW7* which controls GL and GW [11, 12]. *qTGW7*/*qGL7* was at the same position as the gene *FZP* which regulates the number of spikelets per panicle, GL, and TGW [35]. *qGW8*/*qLWR8* was mapped close to *GW8* which controls grain size and quality [13].

Notably, all the five cloned genes were detected in the whole population and multiple subgroups except *GW8*, which was only detected in the *aus* subgroup, suggesting that functionally divergent alleles/haplotypes due to abundant SNP variations should exist in these genes. Despite these several important structural and functional features have been revealed for these proteins in grain size regulation [6, 11–13, 34, 35, 43], it is still of great significance for breeding high yielding and good quality rice varieties to discover the favorable alleles at these loci in the different subgroups. In this study, haplotype analyses of *GS3* has successfully identified the G (C) to T (A) substitution at 16,733,441 bp on chromosome 3 which causes premature termination of the GS3 protein and results in long grain [44, 45]. Other than GL, 824 accessions with Hap1 (TATG) of *GS3* also showed significantly higher means for both TGW and RLW than the other haplotypes in the whole population and most subgroups (Additional file 2, Fig. S8). Notably, Hap2 (GCGA) and Hap5 (GCTG) of *GS3* were exclusively present in the *xian* accessions while Hap3 (GATG) was rarely present indicating that *GS3* may be involved in the *xian*–*geng* differentiation in rice (Additional file 2, Fig. S8). For *GW5*, Hap4 (CGCGC) and Hap7 (CGCG-), mainly represented by the tropical *geng* subgroup, showed significantly higher GL, GW, and RLW than the other haplotypes in the whole population (Additional file 2, Fig. S9). A total of 915 accessions with Hap1 (GAGACGAGA) of *GL7* were predominantly assigned to the *xian* subgroups while 475 accessions carrying the Hap2 (ACCGACGAG) of *GL7* were largely in the *geng* subgroups (Additional file 2, Fig. S10). In the whole population, Hap2 of *GL7* showed significantly higher mean TGW, GL, and GW than Hap1 while Hap1 showed significantly higher mean RLW than that of Hap2 (Additional file 2, Fig. S10). For *FZP*, Hap2 (A) displayed a significantly higher mean GL and RLW and significantly lower GW than Hap1 (G) in the whole population (Additional file 2, Fig. S11). Eight haplotypes of *GW8* were classified by eight SNPs (Additional file 2, Fig. S12). A total of 124 accessions belonged to the *geng* type were assigned in Hap4 (TGCGTGTA) which exhibits the highest TGW, GL, and GW in the whole population. So far, these genes have been widely used in breeding programs, mining of their favorable haplotypes/alleles may facilitate the rational design of grain shape and weight.

### New candidate genes and their future application for improvement of grain yield and quality in rice breeding

Of the three major components (panicle number per plant, number of grains per panicle, and grain weight) of rice yield, grain weight is the most stable and heritable trait, which is measured as the TGW. Moreover, three of the main parameters (GL, GW, and RLW) of grain are positively correlated with grain weight [3]. Meanwhile, grain shape is an important quality trait that affects the market value of rice products. In the current study, seven promising genes located in the six new QTNs for grain weight and grain shape were identified using a large natural population with 2,453 accessions (Table 3). For *qRLW1*, *Os01g0171000* encodes a *BRASSINOSTEROID INSENSITIVE* 1 (*BRI1*)-*Associated receptor Kinase* (*BAK*). Several components of the BR signaling pathway in rice, such as *OsBRI1* (*Brassinosteroid*-*Insensitive1*) [46], *OsBAK1* (*BRI1*-*Associated receptor Kinase1*) [47, 48], and *SERK2* [49] have been proven to regulate plant architecture including grain size. *Os03g0186600* underlying *qTGW3.1* is annotated as *OsMDP1* which negatively regulates brassinosteroid (BR) signaling [50]. Several cloned genes, such as *GW5* [34], *GS5* [51], and *GL3.1*/*qGL3* [52] were also suggested to regulate grain size through the BR signalling pathway. Elucidating the molecular mechanisms of *Os01g0171000* and *Os03g0186600* is essential to make certain of their roles in mediating BR signalling and grain size. The candidate gene *Os04g0580700* for *qGL4*/*qRLW4* encodes a rice MADS-box transcription factor *OsMADS17*. Two genes related to grain size, *OsLG3b* (*OsMADS1*) and *FZP*, regulate the expression of *OsMADS17* [53–55], indicating that *Os04g0580700* may have latent impacts on grain shape and weight.

**Table 3:** List of 7 candidate genes associated with grain weight and grain shape

QTN	Candidate gene^a^	Function
*qTGW3.1*	*Os03g0186600*	*OsMDP1*, MADS-box transcription factor
*qTGW9*	*Os09g0544400*	Glutathione S-transferase, C-terminal domain containing protein
*qTGW11*	*Os11g0163600*	OsFBT13-F-box and tubby domain containing protein
*qGL4/qRLW4*	*Os04g0580700*	*OsMADS17*, MADS-box family gene
*qGL10*	*Os10g0399700*	Cystathionine gamma-synthase
	*Os10g0400100*	Methionyl-tRNA synthetase
*qRLW1*	*Os01g0171000*	BRASSINOSTEROID INSENSITIVE 1-associated receptor kinase

^a^Gene names from the Rice Genome Annotation Project Database based on the Nipponbare reference genome IRGSP-1.0

The haplotypes of genes related to grain weight and grain shape and their distribution in different subgroups obtained in this study can provide more options for breeding by molecular design. When combined with the Kompetitive Allele Specific PCR (KASP) technique [56], breeders can efficiently pyramid favorable alleles of multiple genes that control grain weight and grain shape in rice. Furthermore, precise genome-editing techniques in plants are an alternative important tool for molecular plant breeding [57, 58]. The application of the CRISPR/Cas9-derived system on *GW5* has proven that genome-editing can verify the result of GWAS precisely in a short time [34]. Thus, the favorable haplotypes detected in this study and their further verified functional SNPs will provide useful resources for precise gene editing breeding. Here, we listed dozens of elite rice accessions with combinations of favorable haplotypes of the seven new candidate genes and five well-known genes (Additional file 1: Table S6). For instance, ‘KHAO’ NGAW’ (TGW = 42.76 g, GL = 11.33mm), which is from Thailand and belongs to the subtropical *geng* subgroup, carryies the favorable haplotypes of *GS3*, *GW8*, and *Os03g0186600* for TGW and GL. ‘Diandun 502’ (TGW = 34.04 g, RLW = 3.41) and ‘Mengguandamagu’ (TGW = 33.12 g, RLW = 3.39) are both from China and belong to the intermediate *xian* subgroup, with the favorable haplotypes of *GS3*, *Os03g0186600*, *Os09g0544400*, and *Os10g0399700* for TGW and GL. ‘UP15’ (TGW = 32.29 g, RLW = 3.62) is from Japan and belongs to *basmati* subgroup, carrying the favorable haplotypes of *Os01g0171000* and *Os09g0544400* for RLW and TGW. ‘RACHANDRABHOG’ (TGW = 31.36 g, RLW = 3.73) from India belongs to *xian*-2 subgroup, carrying the favorable haplotypes of *GS3*, *FZP*, and *Os01g0171000* for TGW, GL and RLW. These accessions with high TGW (> 30 g) and RLW (> 3.0) could be used as donor parents for rice breeding and as genetic materials for further functional research.

## Conclusions

There was significant variation in grain weight and grain shape among the 12 rice subgroups that allowed the identification of favourable genes and haplotypes influencing these important traits. Six new QTNs (*qTGW3.1*, *qTGW9, qTGW11*, *qGL4/ qRLW4*, *qGL10*, and *qRLW1*) were identified for grain weight and grain shape by GWAS in a large natural population, and seven candidate genes were screened via high-density association and gene-based haplotype analyses. The results enhance our understanding of the genetic basis of grain weight and grain shape in rice and provide valuable information for elucidating the molecular mechanisms underlying these traits. The exploitation of favorable haplotypes and germplasm resources will be useful for improving rice grain yield and grain quality by molecular breeding.

## Methods

### Plant materials

A total of 2,453 accessions (Additional file 1: Table S1) from the 3 K RGP were used to test the association between the SNP genotype and phenotype of grain weight and grain shape. Based on the known population structure [29], these accessions belong to 12 subgroups, including *aus* (182 accessions), *basmati* (61 accessions), intermediate (81), intermediate *geng* (77 accessions), subtropical *geng* (79 accessions), temperate *geng* (260 accessions), tropical *geng* (291 accessions), *xian*-1 A (206 accessions), *xian*-1B (181 accessions), *xian*-2 (246 accessions), *xian*-3 (257 accessions) and intermediate *xian* (532 accessions).

### Field trials and trait measurements

All accessions were grown at Sanya, China (18.3^◦^N, 109.3^◦^E) for three consecutive years from 2015 to 2017. Seeds were surface sterilized and approximately 100 seeds of each accession were sowed on 8 November 2015, 5 November 2016, and 20 November 2017. At 25 days after sowing, the seedlings were transplanted into a three-row plot with 10 individuals in each row at a spacing of 20 cm × 25 cm. Field trials were carried out following a randomized complete block design with two replications in each year. The field management followed the local farmers’ standard practices. At the full-ripe stage (about 40 days after flowering), seeds of eight plants in the middle of each plot were bulk harvested and air-dried in the screen house until reaching a constant seed weight.

The method of trait measurement using the automatic seed counting and analyzing instrument (Model SC-G, Hangzhou Wanshen Detection Technology Co., Ltd., Hangzhou, China, http://www.wseen.com/) has been described before [6, 59]. Briefly, at least 300 fully-filled seeds of each accession were scattered evenly on a flat-bed scanner (30 cm × 25 cm) and imaged with a high-resolution camera. The seeds number, GL in mm, GW in mm and RLW were calculated by analyzing the image via the grain analyzer software using the rice model. Then the weight of these seeds was measured using a high precision electronic balance (1/1000, APTP456 series) and the TGW in grams was subsequently calculated. The scanner was calibrated with a calibration target before each measurement.

### Statistical analyses of phenotypic data

The R package ‘lme4’ [60] was used to obtain the best linear unbiased estimate (BLUE) for each genotype-environment combination and variance components using linear mixed models. Genotype and replication were treated as a fixed effect and a random effect for single-environment analysis, respectively. The BLUEs of each year were subsequently calculated and used for analysis of variance (ANOVA) and GWAS. Variance components were estimated using multi-environment analysis with genotype treated as a fixed effect while environment, replicate within an environment and genotype-by-environment interaction treated as random effects. The narrow-sense heritability (*h*^*2*^) was estimated as: *h*^*2*^ = V_g_/(V_g_ +V_gei_/s + V_e_/sr), where V_g_, V_gei_, and V_e_ are the variance contributed by genotype, genotype-by-environment interaction, and residual error, respectively, while s is the number of environments and r is the number of replicates. Then, the BLUEs of three years for each accession were calculated and used for computing the Pearson’s correlation coefficients among traits with the ‘Hmisc’ package in R. One-way ANOVA followed by Duncan’s multiple range test were used for statistical comparisons across multiple means of TGW, GL, GW, and RLW among the 12 rice subgroups by the ‘agricolae’ package in R.

### Genome-wide association mapping

We conducted association studies to identify genome-wide signals associated with grain weight and grain shape in the whole population and five major subgroups, *aus*, *basmati*, *xian* (including *xian*-1A, *xian*-1B, *xian*-2, *xian*-3, and intermediate *xian*), temperate *geng*, and tropical *geng*, to minimize the impact of population structure on the power of GWAS. The 4.8 M SNP dataset of 3K RGP was downloaded from the Rice SNP-Seek Database [61]. After filtering out SNPs with a missing rate of more than 20 % or with a minor allele frequency (MAF) less than 5 % using PLINK software [62] with the parameter ‘--geno 0.2’, and ‘ --maf 0.05’, a total of 3,343,302, 2,240,362, 865,777, 1,548,277, 1,884,822,and 1,728,815 SNPs were retained for GWAS in the whole population, *xian*, temperate *geng*, tropical *geng*, *aus* and *basmati* subgroups, respectively.

A single-locus GWAS was performed with a linear mixed model to determine the association between each SNP and the measured phenotypes by an efficient mixed-model analysis with EMMA eXpedited (EMMAX) software [63]. The kinship matrix was generated using an identical-by-state matrix based on the subset of genome-wide SNP data with the ‘indep-pairwise 50 10 0.1’ parameter in PLINK to account for the relatedness among accessions. The first three principal components were used as covariates (Q-matrix) to control population structure. The effective number of independent markers (N) was calculated using the GEC software [64] and suggestive *P*-value thresholds of association (1/N) were 1.76E-6, 2.40E-6, 1.03E-5, 5.63E-6, 4.08E-6, and 5.93E-6 for the whole population, *xian*, temperate *geng*, tropical *geng*, *aus*, and *basmati*, respectively. These suggestive *P*-value thresholds were used to claim significant SNP-trait associations/QTNs for the whole population and the five subgroups, respectively. Manhattan and quantile-quantile (Q–Q) plots of GWAS were created by the R package ‘qqman’ [65].

### Linkage Disequilibrium (LD) decay estimates

We calculated *r*^*2*^, an estimation of LD, using PLINK software with the parameter ‘–r2 –ld-window-kb 1000 –ld-window 99999 –ld-window-r2 0’. The LD decay rate was measured as the chromosomal distance at which the average *r*^*2*^ dropped to half of its maximum value. The LD block harboring significant trait-associated SNPs was defined as the candidate region for each QTN, and the SNP with the minimum *P*-value within an LD block was considered as the lead SNP.

### Candidate gene identification

High-density association and gene-based haplotype analyses were carried out to detect candidate genes for stable QTNs that could be detected in at least two years and were newly identified in our study. The following five steps were conducted to identify candidate genes for each QTN: 1) all available SNPs located in the QTN region were acquired from 29 M SNPs data of 3K RGP in the Rice SNP-Seek Database [61]; 2) the SNPs were filtered with the aforementioned parameters and the remaining high-quality SNPs were used to perform high-density association analyses through EMMAX software; 3) the gene with the most significant hit within a local LD block constructed around the stable QTNs was screened as the candidate gene. The R package ‘LDheatmap’ [66] was used to draw the heatmap of pairwise LDs; 4) the annotated genes from the Rice Genome Annotation Project Database (RAP-DB) [67] that harbor the significant SNPs were selected as the candidate genes; 5) gene-based haplotype analysis was carried out for each candidate gene. The SNPs within 2 kb of the upstream promoter region, 3’ untranslated region (UTR), and 5’ UTR, as well as non-synonymous SNPs in the coding regions of a candidate gene, were concatenated as the haplotype. Only major haplotypes of each candidate gene carried by at least 15 accessions in the whole population and at least 5 accessions in subgroups were used for multiple comparisons. BLUEs of three years were applied for the multiple comparisons of the major haplotypes. Duncan’s multiple comparison tests (5 % significance level) followed by one-way ANOVA were completed with the ‘agricolae’ package in R.

## Supplementary Informationok

**Additional file 1: Table S1.** Summary of 2,453 rice accessions used in our study. **Table S2.** Analysis of the variance in traits related to grain weight and grain shape. **Table S3.** Performance of grain weight and grain shape in different subgroups. **Table S4.** ANOVA for haplotypes of 7 candidate genes associated with grain weight and grain shape in the whole population and each subgroup. **Table S5.** Haplotype analyses of candidate genes associated with grain weight and grain shape. **Table S6.** Elite accessions with high TGW and RLW. **Table S7.** Raw phenotyping data of 2453 accessions collected in 2015, 2016 and 2017.

**Additional file 2: Figure S1.** Comparison of LD decay in the whole population and five major subgroups. Y axis was the average *r*^2^ values of each 1 Mb region and X axis was physical distance between markers in unit of Mb. **Figure S2.** A‒F. Manhattan and QQ plots for TGW, GL, GW and RLW of the whole population (S2A), aus subgroup (S2B), basmati subgroup (S2C), *xian*subgroup (S2D), temperate *geng*subgroup (S2E), tropical *geng*subgroup (S2F) in 2015. **Figure S3.** A‒F. Manhattan and QQ plots for TGW, GL, GW and RLW of the whole subgroup (S3A), aus subgroup (S3B), basmati subgroup (S3C), *xian* subgroup (S3D), temperate *geng* subgroup (S3E), tropical *geng* subgroup (S3F) in 2016.**Figure S4.** A‒F. Manhattan and QQ plots for TGW, GL, GW and RLW of the whole subgroup (S4A), aus subgroup (S4B), basmati subgroup (S4C), *xian* subgroup (S4D), temperate *geng* subgroup (S4E), tropical *geng* subgroup (S4F) in 2017. **Figure S5.** (a) High-density association analysis of *qTGW9* in 2015, 2016, and 2017. The solid line indicates the threshold to determine significant SNP. (b-c) Gene structural of candidate gene *Os09g0544400* and haplotype analysis for TGW in the whole population and the 12 subgroups. Characters on top of boxplots indicate significant differences based on Duncan’s multiple comparison tests (*P* < 0.05). **Figure S6.** High-density association analysis of *qTGW11* in 2015, 2016, and 2017. The solid line indicates the threshold to determine significant SNP. (b-c) Gene structural of candidate gene*Os11g0163600* and haplotype analysis for TGW in the whole population and the 12 subgroups. Characters on top of boxplots indicate significant differences based on Duncan’s multiple comparison tests (*P* < 0.05). **Figure S7.** (a) High-density association analysis of *qGL10* in 2015, 2016, and 2017. The solid line indicates the threshold to determine significant SNP. (b‒c) Gene structural of candidate genes *Os10g0399700* and *Os10g0400100*, and haplotype analyses for GL in the whole population and the 12 subgroups. Characters on top of boxplots indicate significant differences based on Duncan’s multiple comparison tests (*P* < 0.05). **Figure S8.** Haplotypes analysis of*GS3*.Error bars, standard error of the mean (SEM). Characters on top of boxplots indicate significant differences based on Duncan’s multiple comparison tests (*P*< 0.05). **Figure S9.** Haplotypes analysis of *GW5*.Error bars, standard error of the mean (SEM). Characters on top of boxplots indicate significant differences based on Duncan’s multiple comparison tests (*P* < 0.05). **Figure S10.** Haplotypes analysis of*GL7*.Error bars, standard error of the mean (SEM). Characters on top of boxplots indicate significant differences based on Duncan’s multiple comparison tests (*P*< 0.05). **Figure S11.** Haplotypes analysis of *FZP*.Error bars, standard error of the mean (SEM). Characters on top of boxplots indicate significant differences based on Duncan’s multiple comparison tests (*P* < 0.05). **Figure S12.** Haplotypes analysis of*GW8*.Error bars, standard error of the mean (SEM). Characters on top of boxplots indicate significant differences based on Duncan’s multiple comparison tests (*P*< 0.05).

## Data Availability

The 4.8 M and 29 M SNP datasets used and/or analyzed during the current study are available from the Rice SNP-Seek Database (https://snp-seek.irri.org/_download.zul). The phenotype dataset used during the current study is provided in the Addational file 1: Table S7.

## Abbreviations

TGW: Thousand grain weight
GL: Grain length
GW: Grain width
RLW: Ratio of grain length to grain width
3 K RGP: 3,000 Rice Genome Project
ANOVA: Analysis of variance
GWAS: Genome-wide association study
QTL: Quantitative trait locus/loci
QTNs: Quantitative trait nucleotides
SNP: Single nucleotide polymorphism
MAF: Minor allele frequency
kb: Kilobyte
Mb: Megabyte
LD: Linkage disequilibrium
CDS: Coding DNA sequence
UTR: Untranslated region
