# Supplementary material for: Identification and allele mining of new candidate genes underlying rice grain weight and grain shape by genome-wide association study
Source: BMC Genomics. 2021 Aug 6;22:602. doi: 10.1186/s12864-021-07901-x (PMC8349016; doi:10.1186/s12864-021-07901-x)
Supplement: Supplementary file 2 — Additional file 2: Figure S1. Comparison of LD decay in the whole population and five major subgroups. Y axis was the average r2 values of each 1 Mb region and X axis was physical distance between markers in unit of Mb. Figure S2. A‒F. Manhattan and QQ plots for TGW, GL, GW and RLW of the whole population (S2A), aus subgroup (S2B), basmati subgroup (S2C), xiansubgroup (S2D), temperate gengsubgroup (S2E), tropical gengsubgroup (S2F) in 2015. Figure S3. A‒F. Manhattan and QQ plots for TGW, GL, GW and RLW of the whole subgroup (S3A), aus subgroup (S3B), basmati subgroup (S3C), xian subgroup (S3D), temperate geng subgroup (S3E), tropical geng subgroup (S3F) in 2016.Figure S4. A‒F. Manhattan and QQ plots for TGW, GL, GW and RLW of the whole subgroup (S4A), aus subgroup (S4B), basmati subgroup (S4C), xian subgroup (S4D), temperate geng subgroup (S4E), tropical geng subgroup (S4F) in 2017. Figure S5. (a) High-density association analysis of qTGW9 in 2015, 2016, and 2017. The solid line indicates the threshold to determine significant SNP. (b-c) Gene structural of candidate gene Os09g0544400 and haplotype analysis for TGW in the whole population and the 12 subgroups. Characters on top of boxplots indicate significant differences based on Duncan’s multiple comparison tests (P < 0.05). Figure S6. High-density association analysis of qTGW11 in 2015, 2016, and 2017. The solid line indicates the threshold to determine significant SNP. (b-c) Gene structural of candidate geneOs11g0163600 and haplotype analysis for TGW in the whole population and the 12 subgroups. Characters on top of boxplots indicate significant differences based on Duncan’s multiple comparison tests (P < 0.05). Figure S7. (a) High-density association analysis of qGL10 in 2015, 2016, and 2017. The solid line indicates the threshold to determine significant SNP. (b‒c) Gene structural of candidate genes Os10g0399700 and Os10g0400100, and haplotype analyses for GL in the whole population and the 12 subgroups. Charac [file 12864_2021_7901_MOESM2_ESM.pptx]

## Slide 1
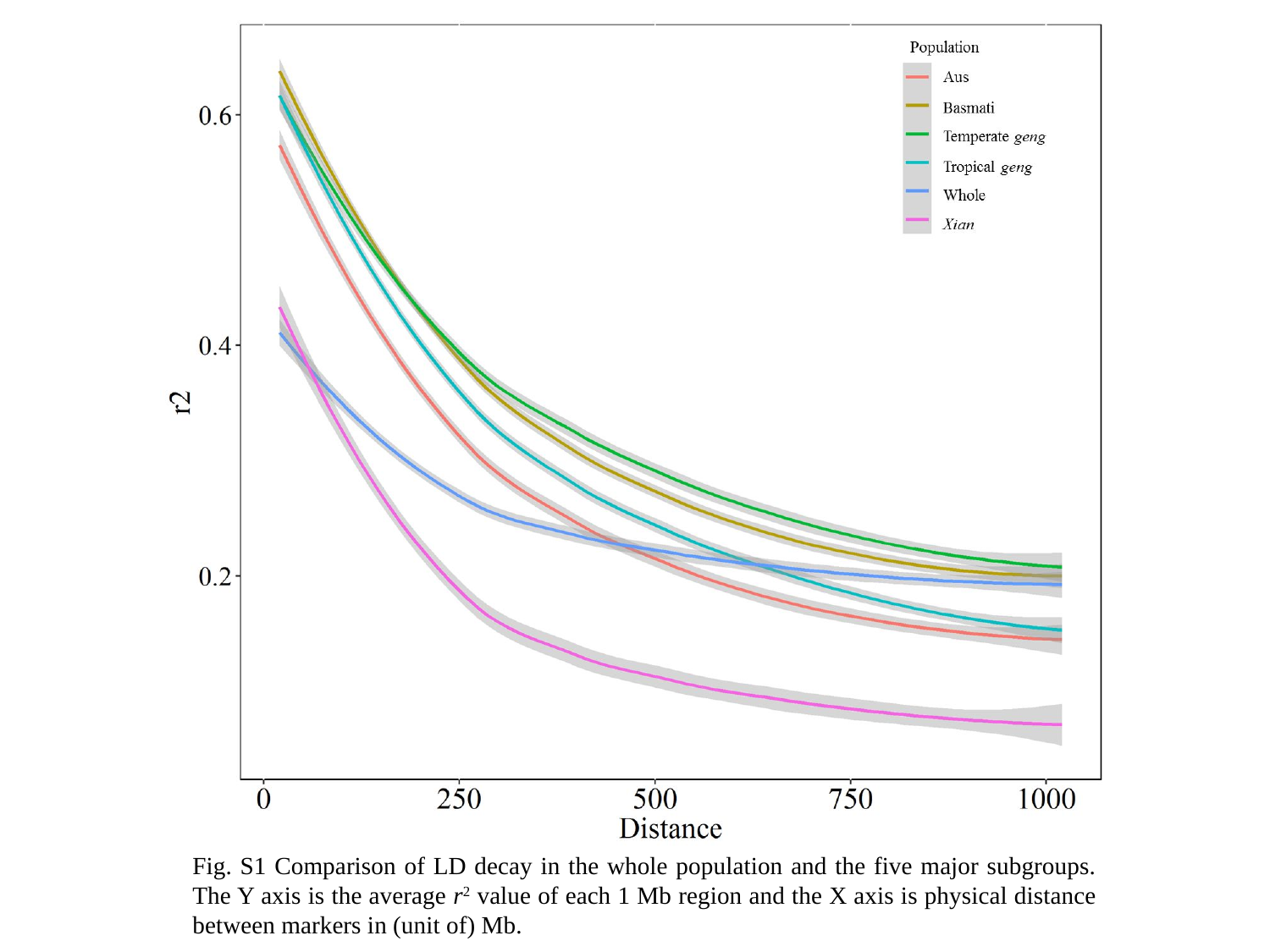

Fig. S1 Comparison of LD decay in the whole population and the five major subgroups. The Y axis is the average r2 value of each 1 Mb region and the X axis is physical distance between markers in (unit of) Mb.

## Slide 2
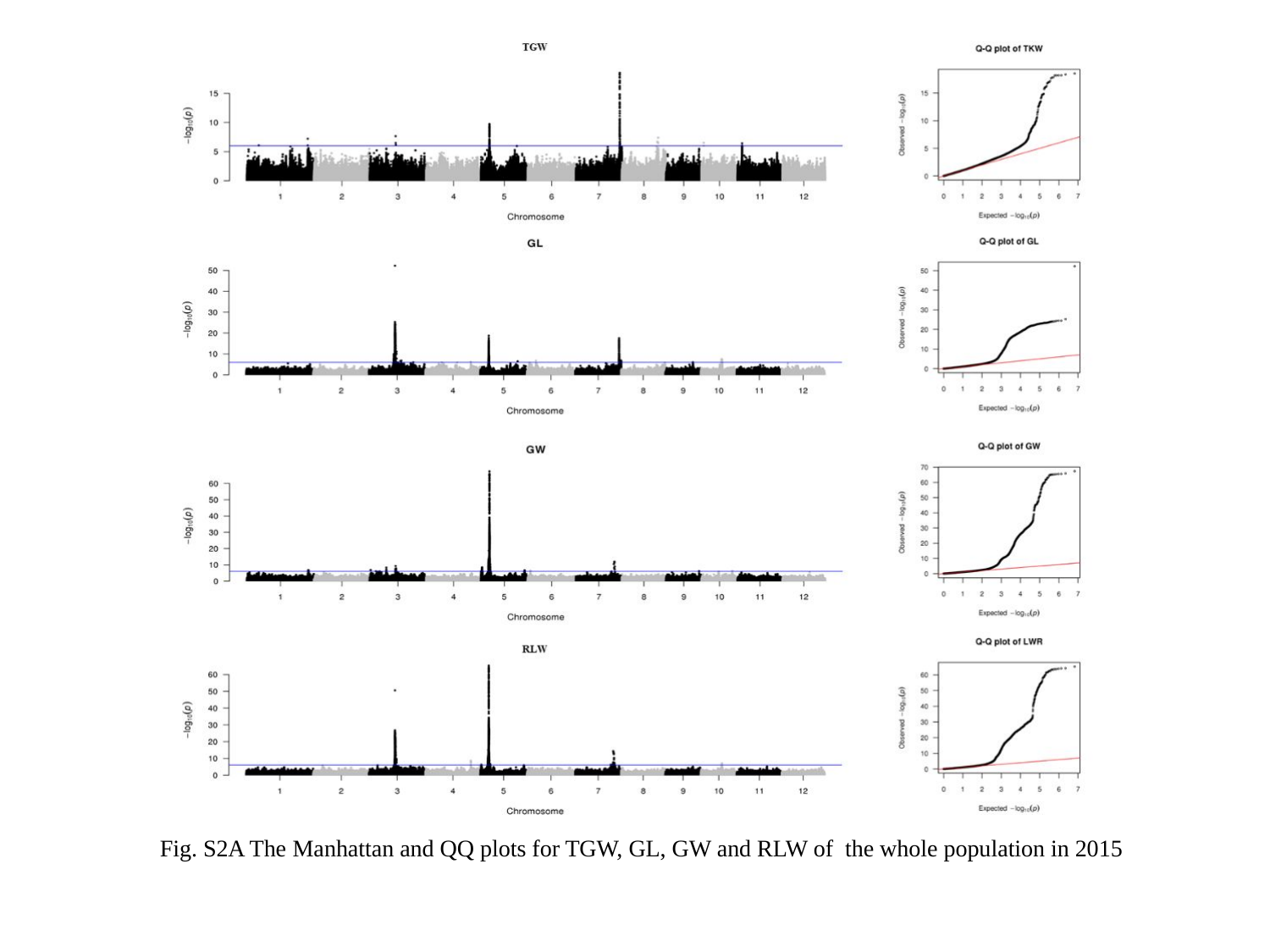

Fig. S2A The Manhattan and QQ plots for TGW, GL, GW and RLW of the whole population in 2015

## Slide 3
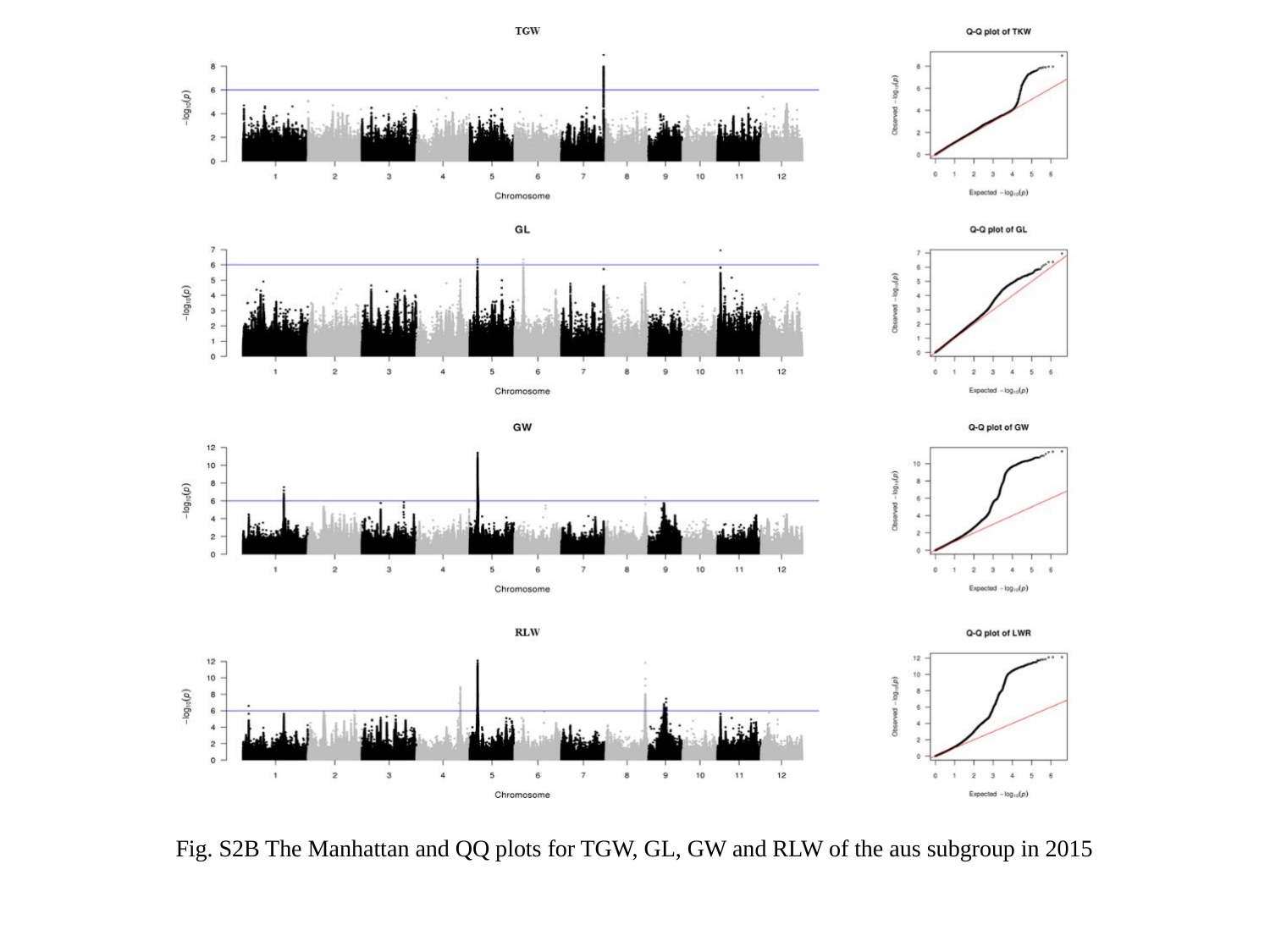

Fig. S2B The Manhattan and QQ plots for TGW, GL, GW and RLW of the aus subgroup in 2015

## Slide 4
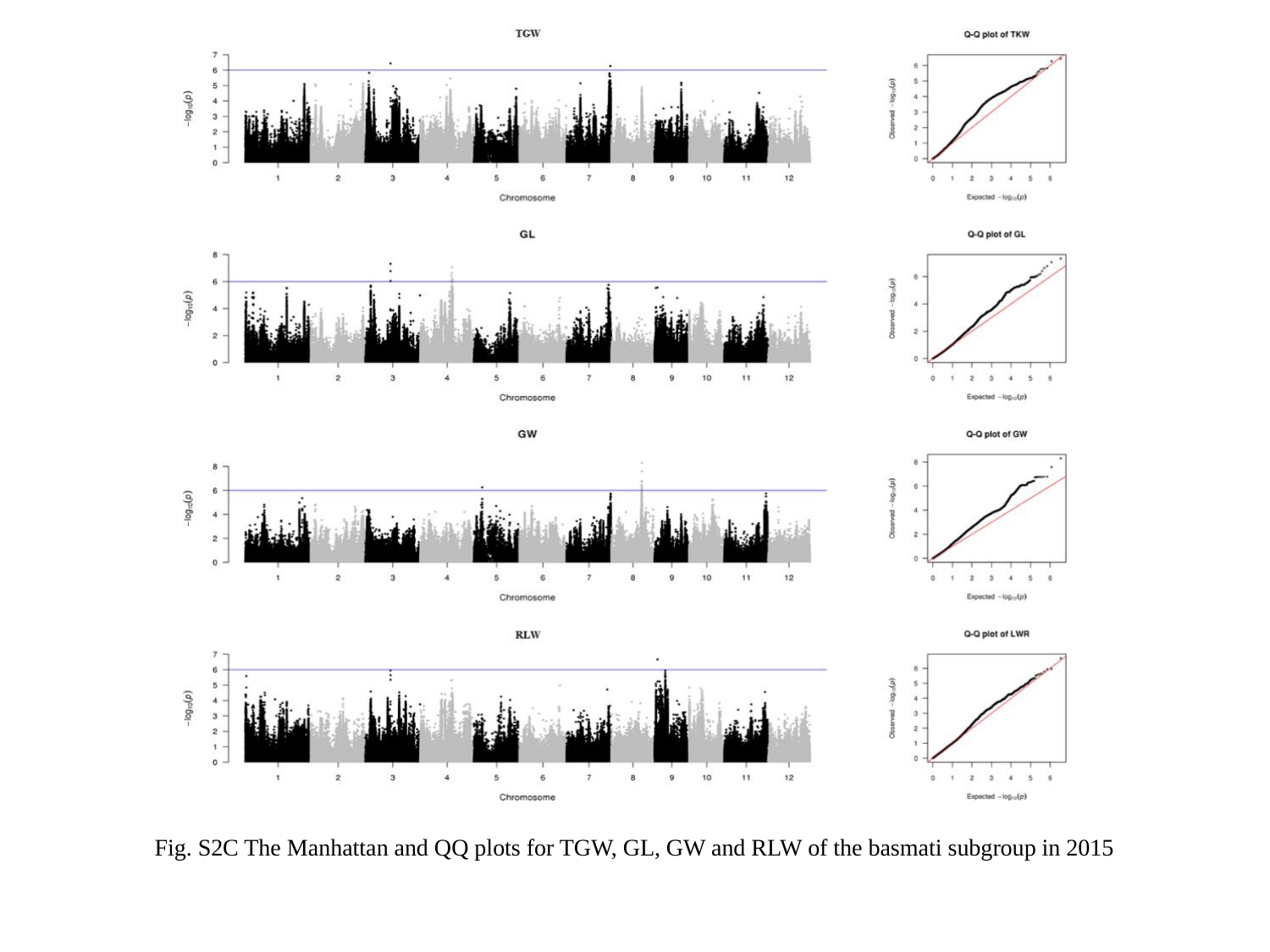

Fig. S2C The Manhattan and QQ plots for TGW, GL, GW and RLW of the basmati subgroup in 2015

## Slide 5
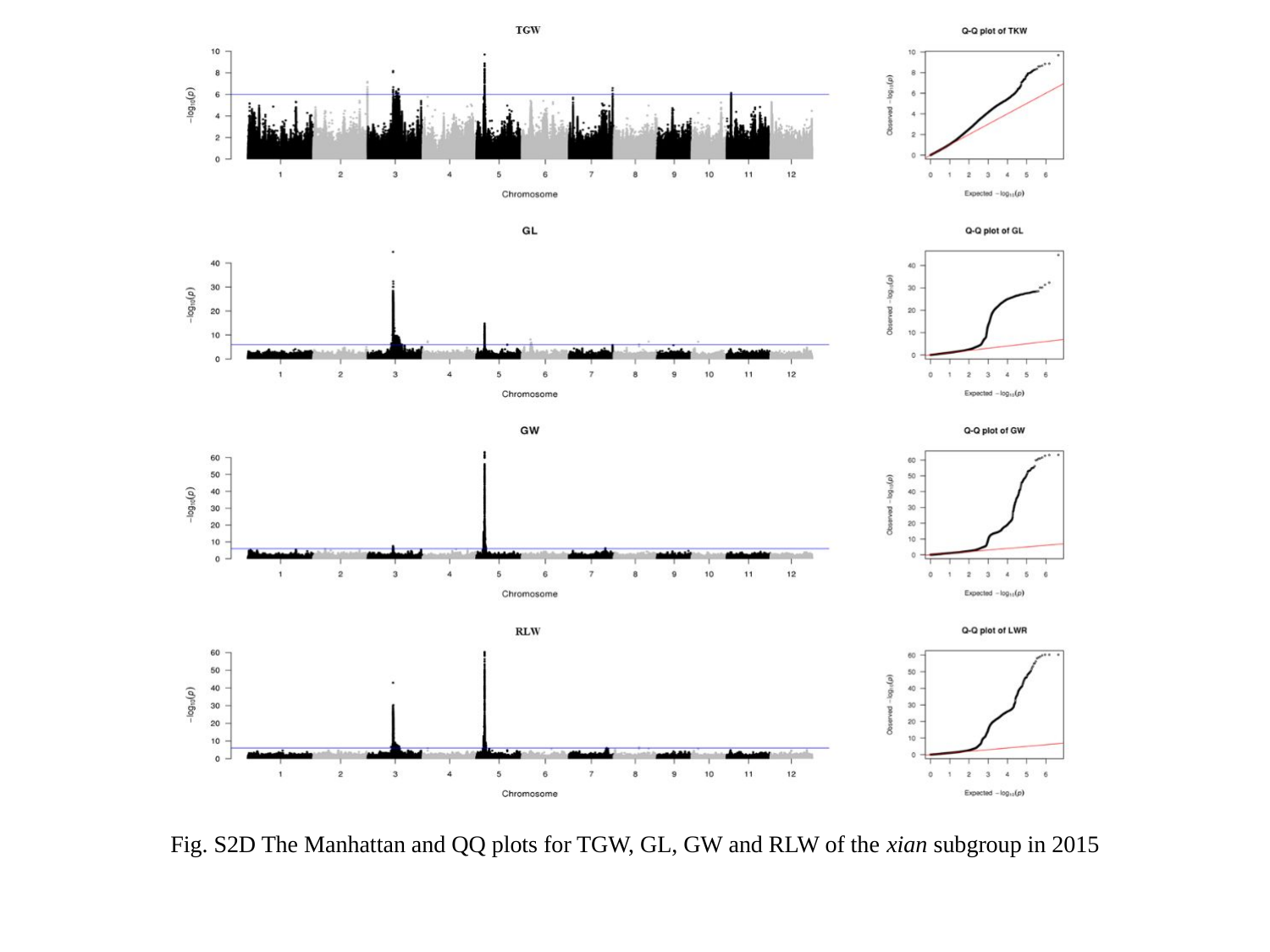

Fig. S2D The Manhattan and QQ plots for TGW, GL, GW and RLW of the xian subgroup in 2015

## Slide 6
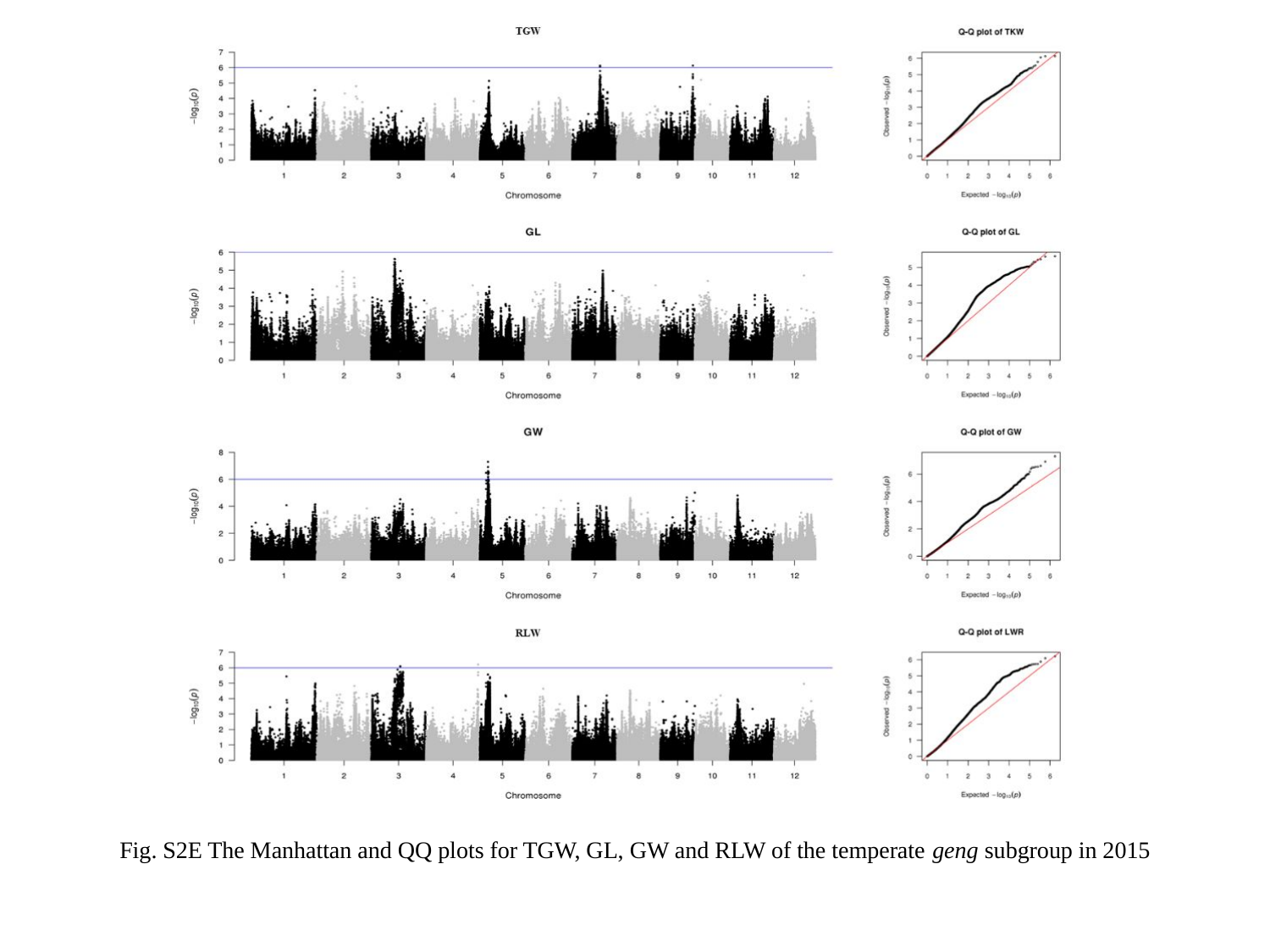

Fig. S2E The Manhattan and QQ plots for TGW, GL, GW and RLW of the temperate geng subgroup in 2015

## Slide 7
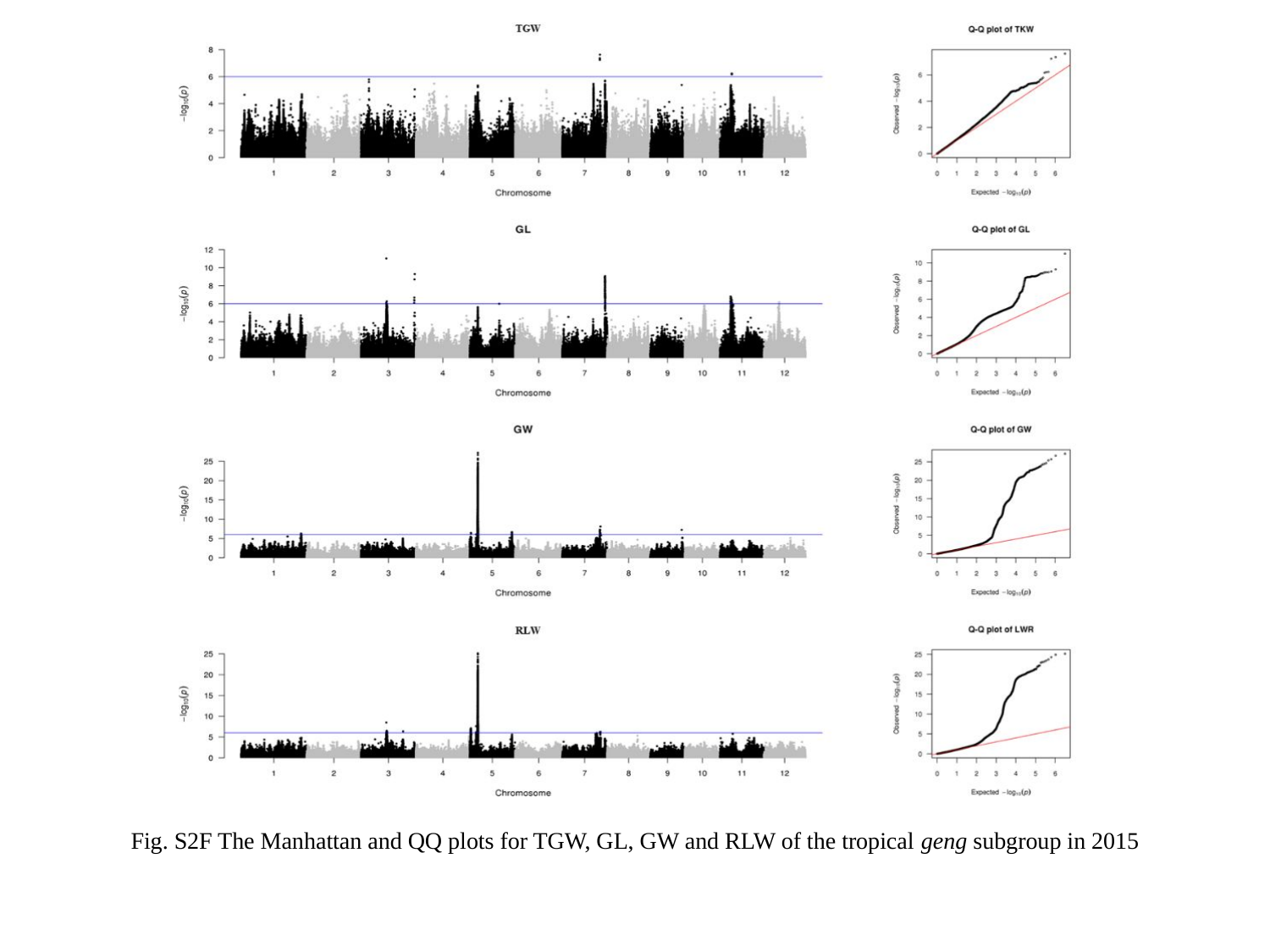

Fig. S2F The Manhattan and QQ plots for TGW, GL, GW and RLW of the tropical geng subgroup in 2015

## Slide 8
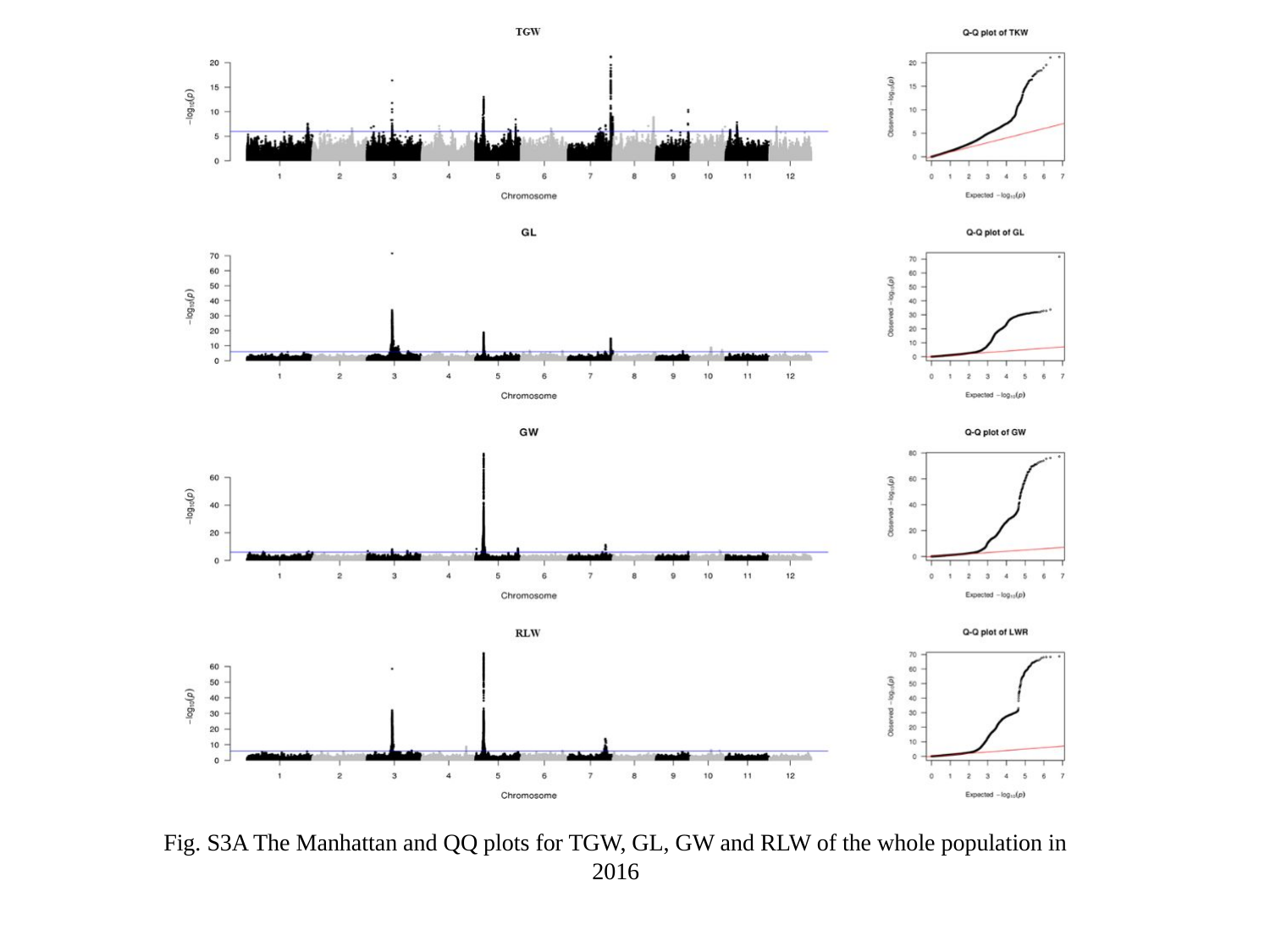

Fig. S3A The Manhattan and QQ plots for TGW, GL, GW and RLW of the whole population in 2016

## Slide 9
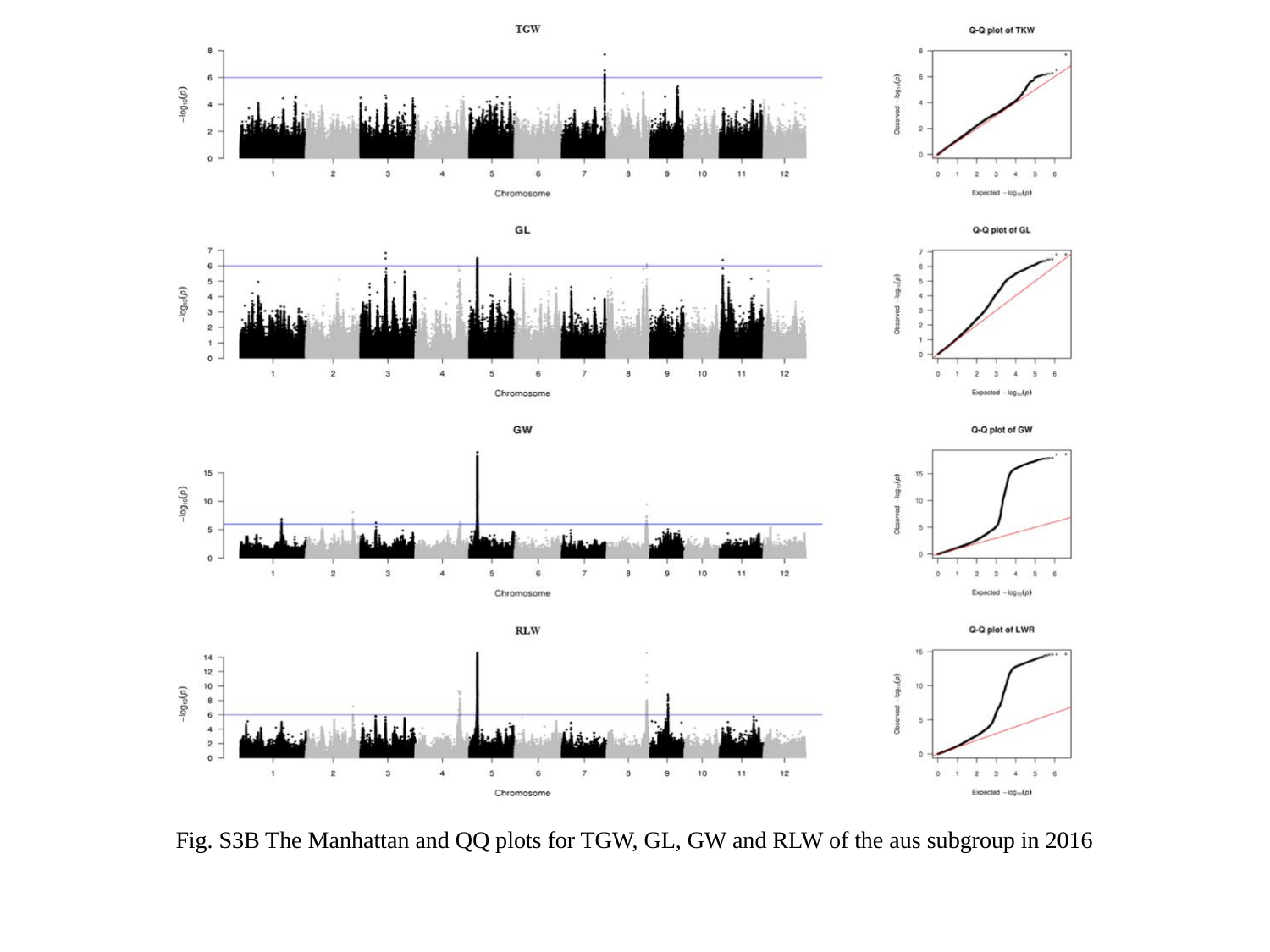

Fig. S3B The Manhattan and QQ plots for TGW, GL, GW and RLW of the aus subgroup in 2016

## Slide 10
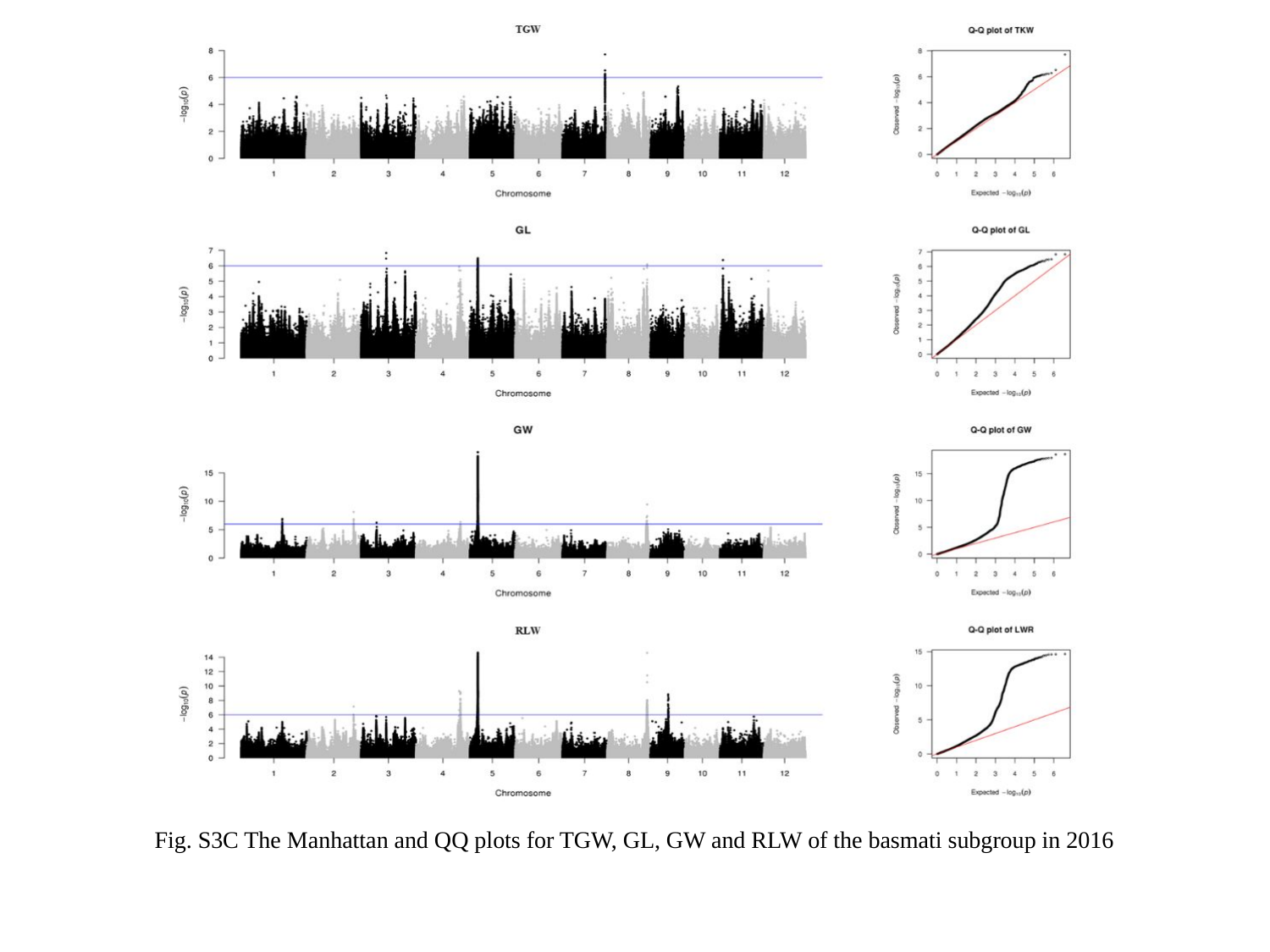

Fig. S3C The Manhattan and QQ plots for TGW, GL, GW and RLW of the basmati subgroup in 2016

## Slide 11
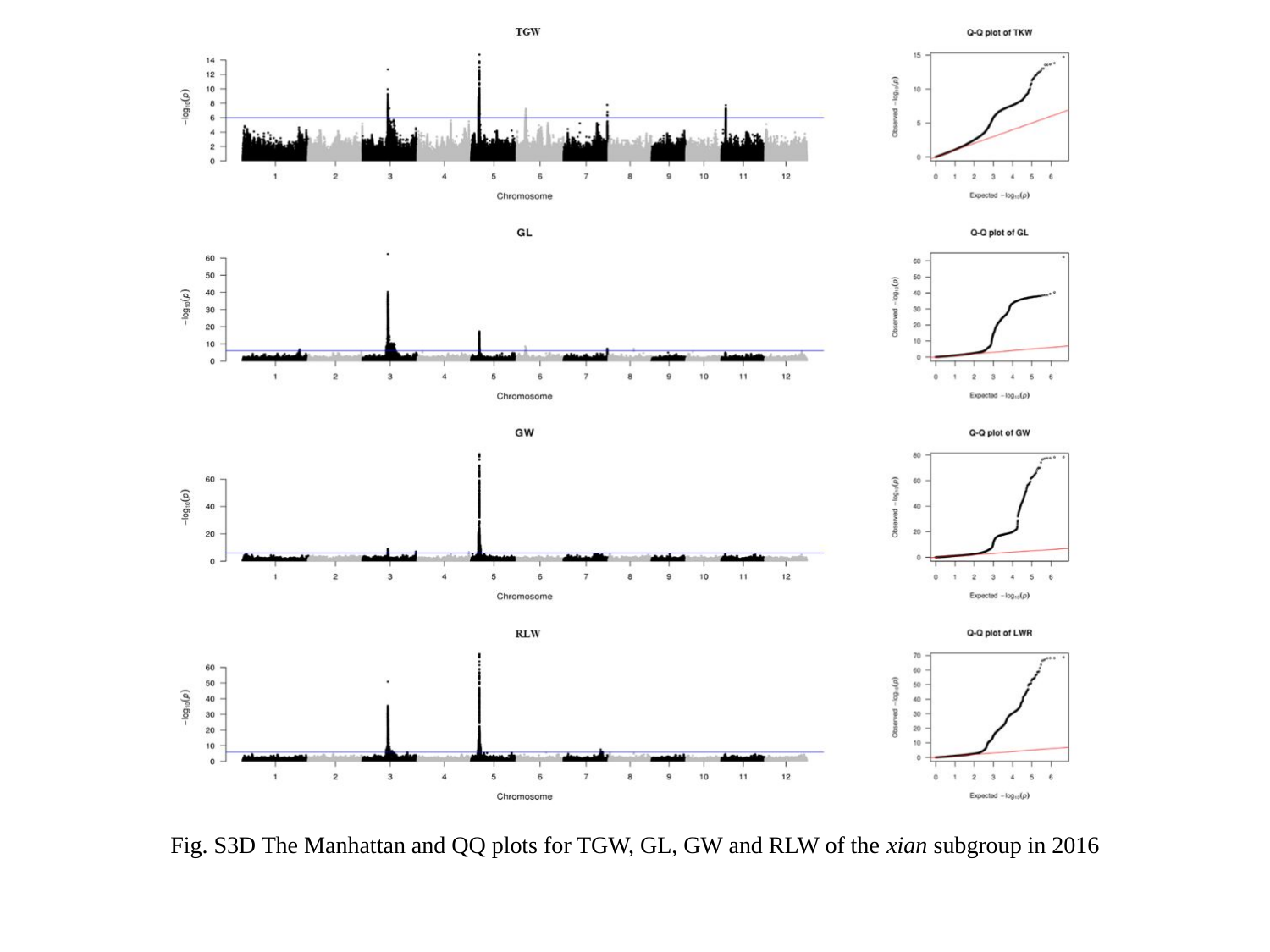

Fig. S3D The Manhattan and QQ plots for TGW, GL, GW and RLW of the xian subgroup in 2016

## Slide 12
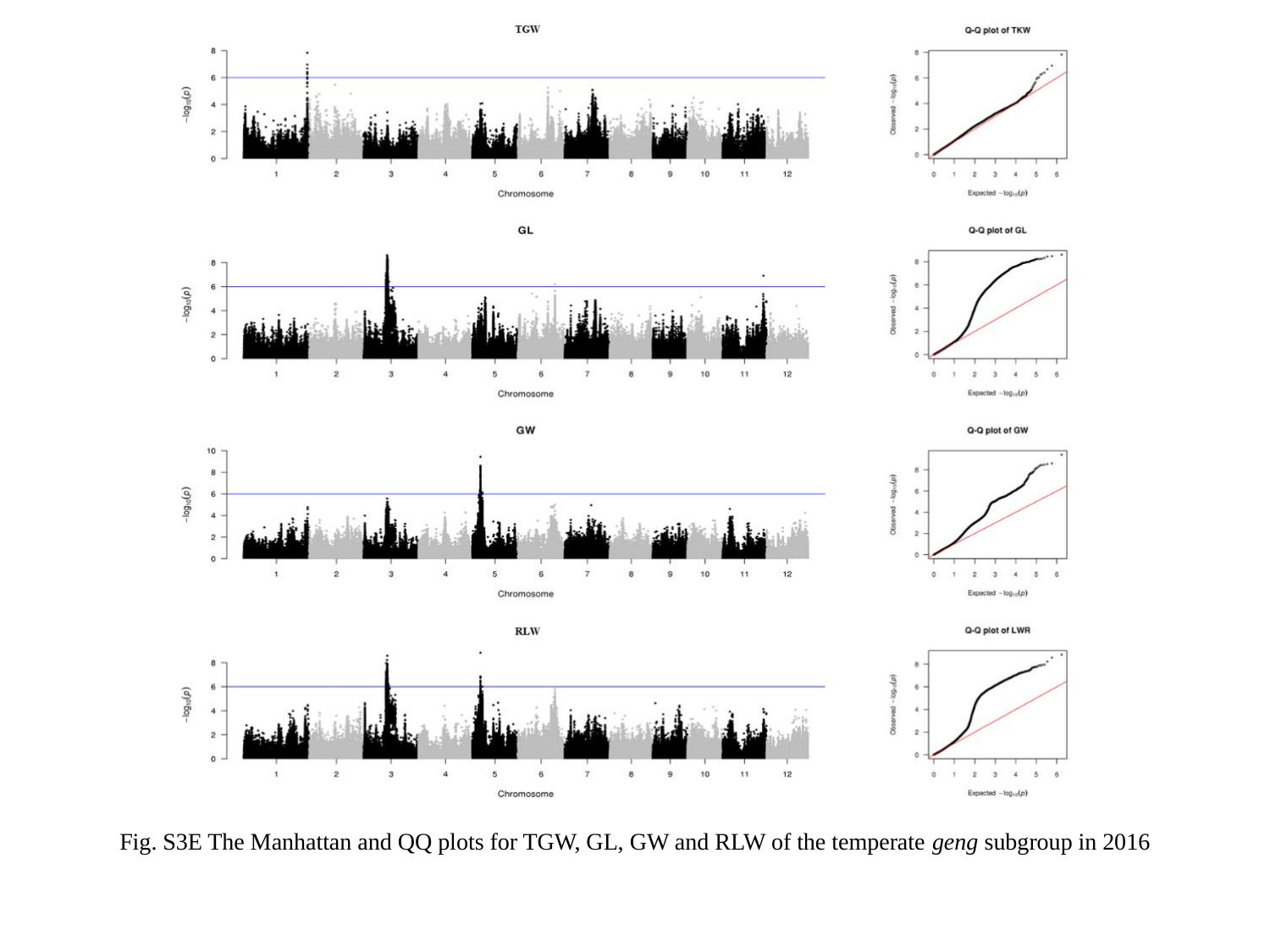

Fig. S3E The Manhattan and QQ plots for TGW, GL, GW and RLW of the temperate geng subgroup in 2016

## Slide 13
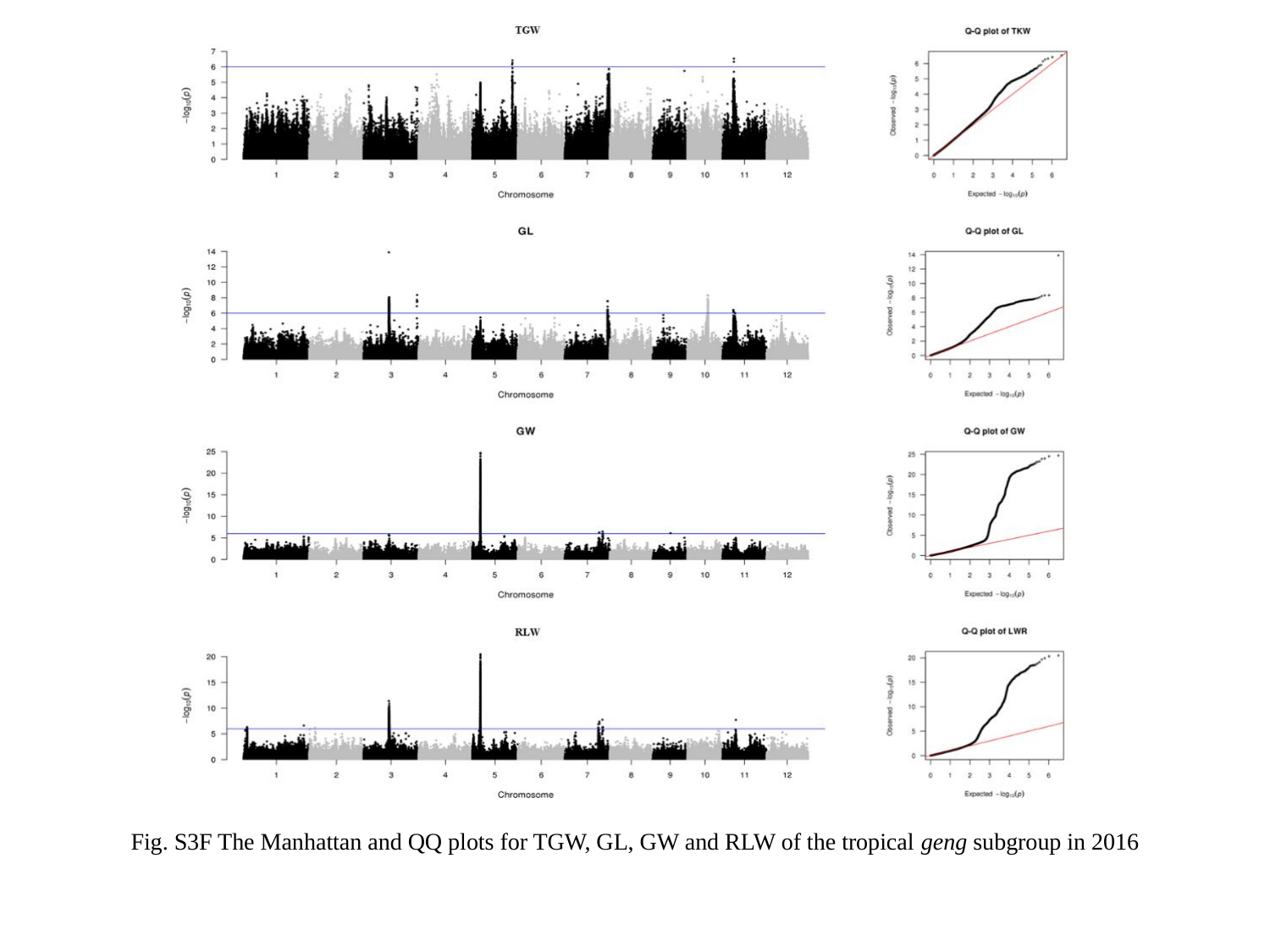

Fig. S3F The Manhattan and QQ plots for TGW, GL, GW and RLW of the tropical geng subgroup in 2016

## Slide 14
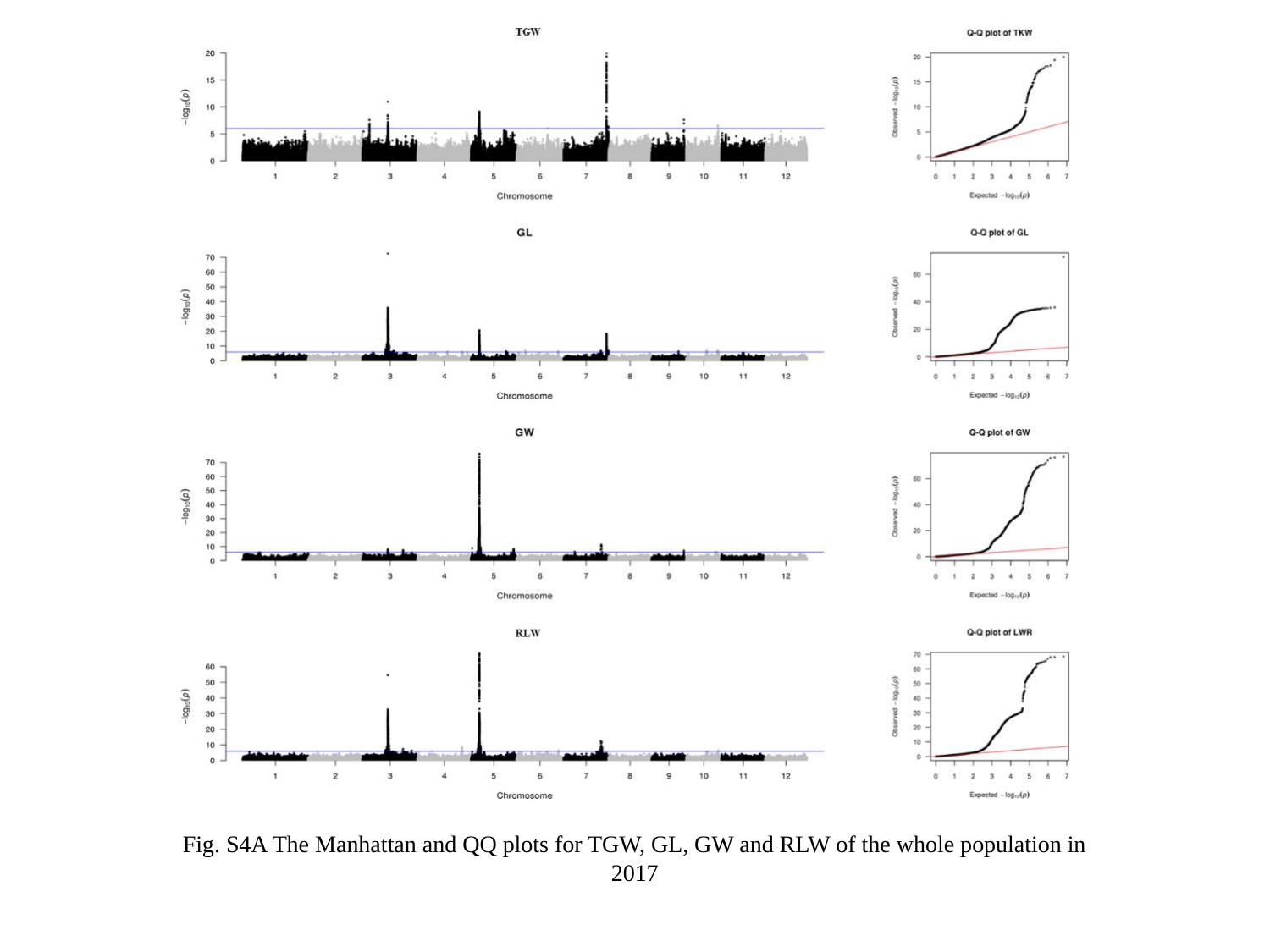

Fig. S4A The Manhattan and QQ plots for TGW, GL, GW and RLW of the whole population in 2017

## Slide 15
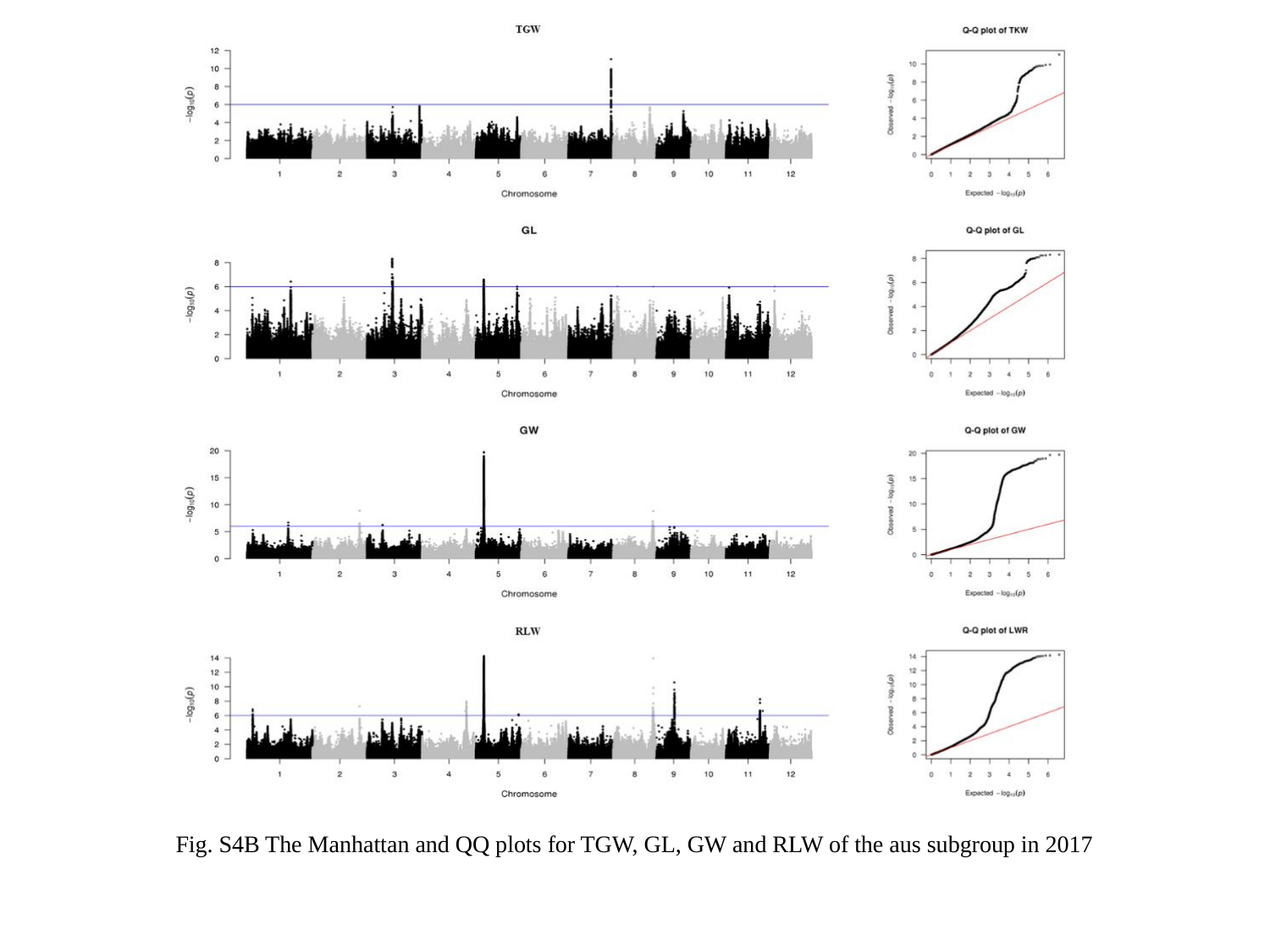

Fig. S4B The Manhattan and QQ plots for TGW, GL, GW and RLW of the aus subgroup in 2017

## Slide 16
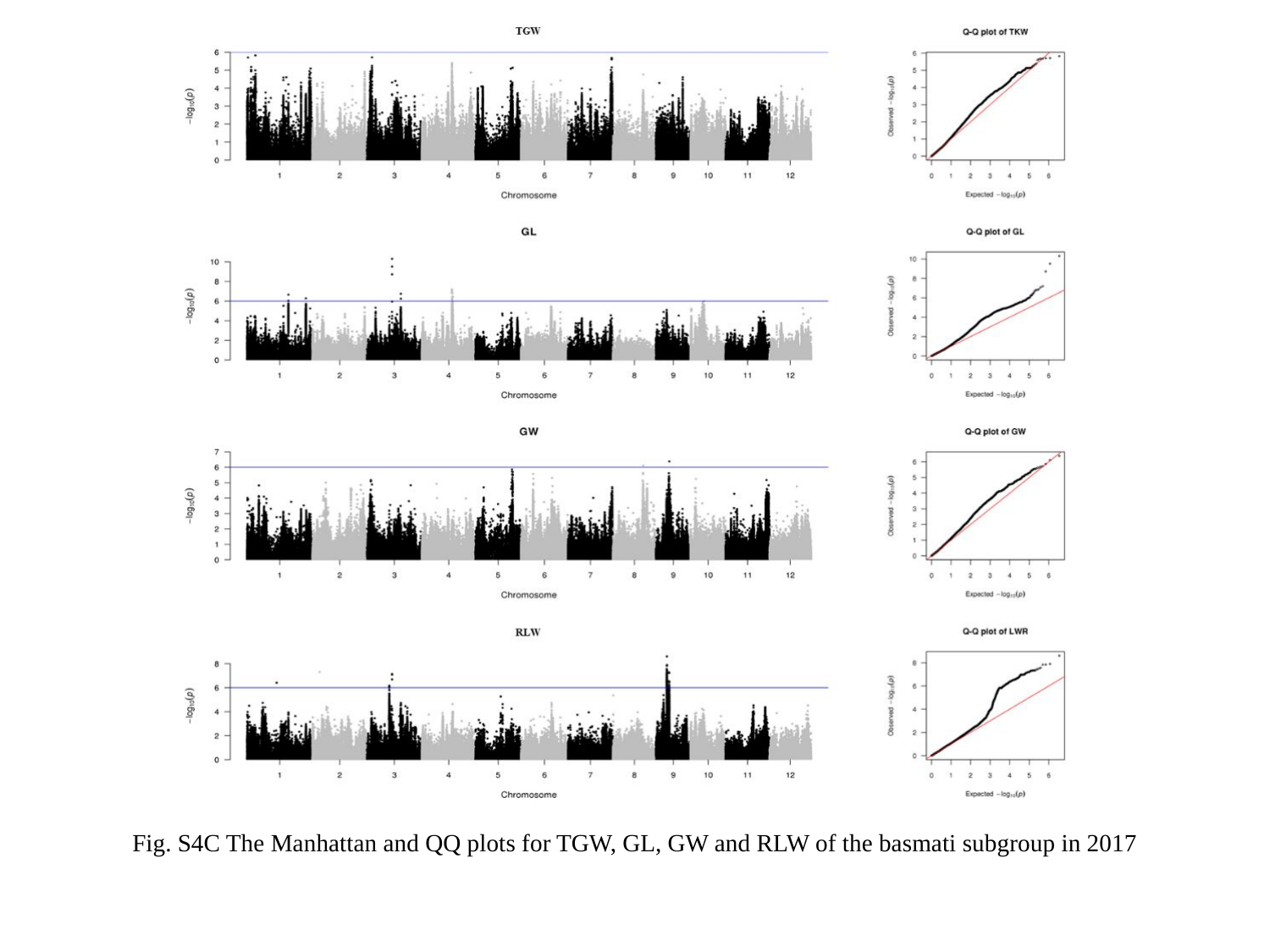

Fig. S4C The Manhattan and QQ plots for TGW, GL, GW and RLW of the basmati subgroup in 2017

## Slide 17
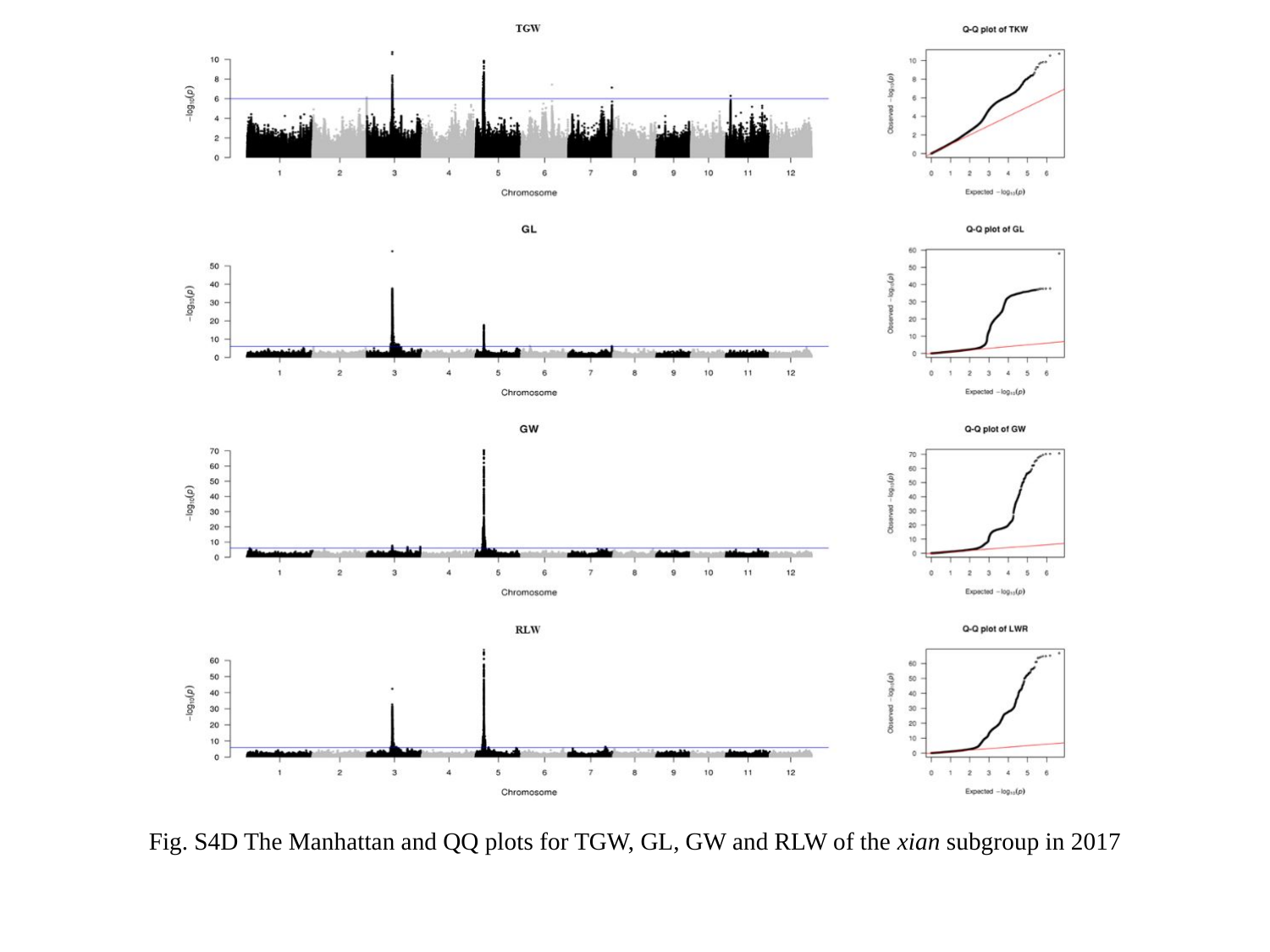

Fig. S4D The Manhattan and QQ plots for TGW, GL, GW and RLW of the xian subgroup in 2017

## Slide 18
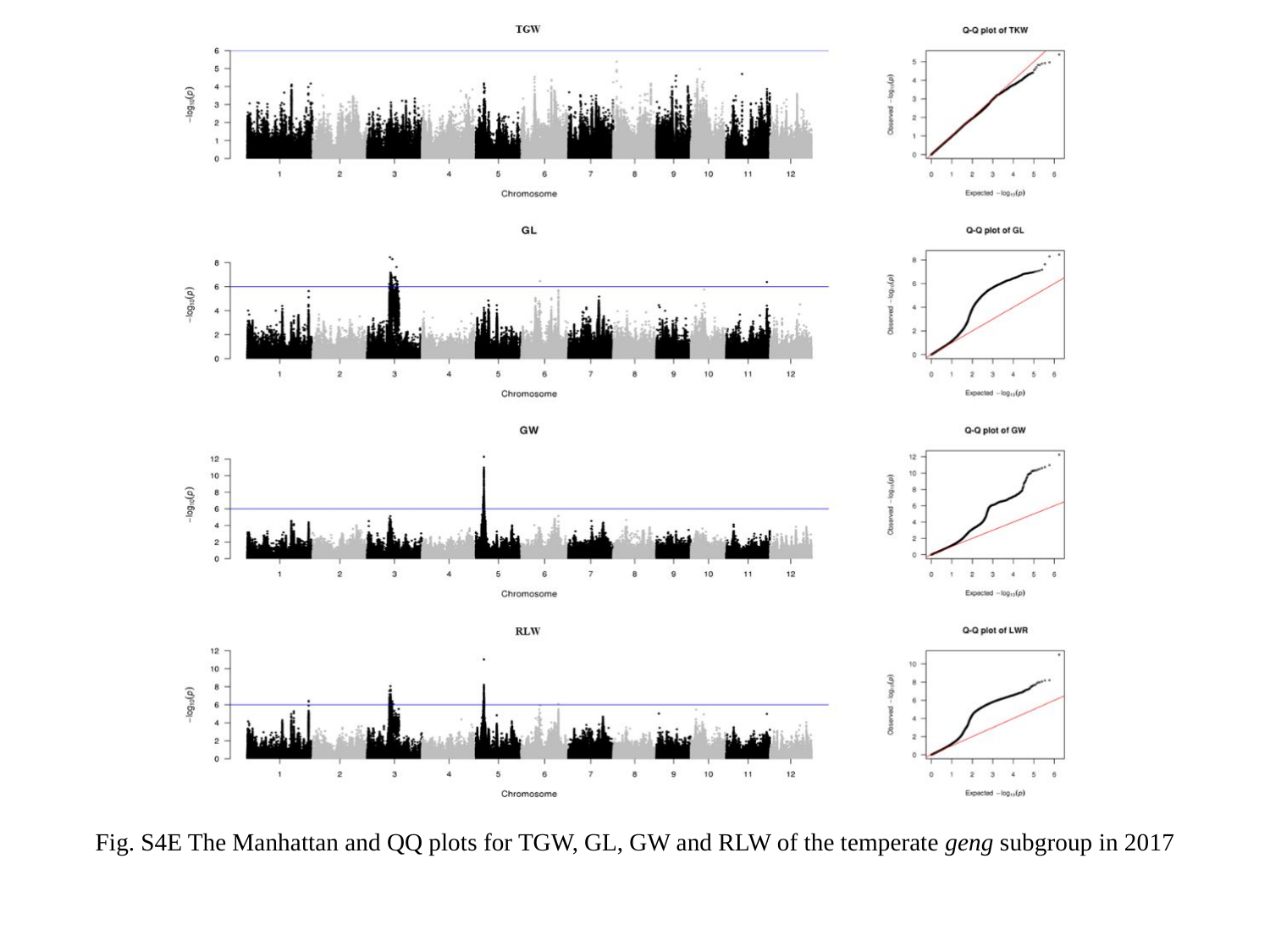

Fig. S4E The Manhattan and QQ plots for TGW, GL, GW and RLW of the temperate geng subgroup in 2017

## Slide 19
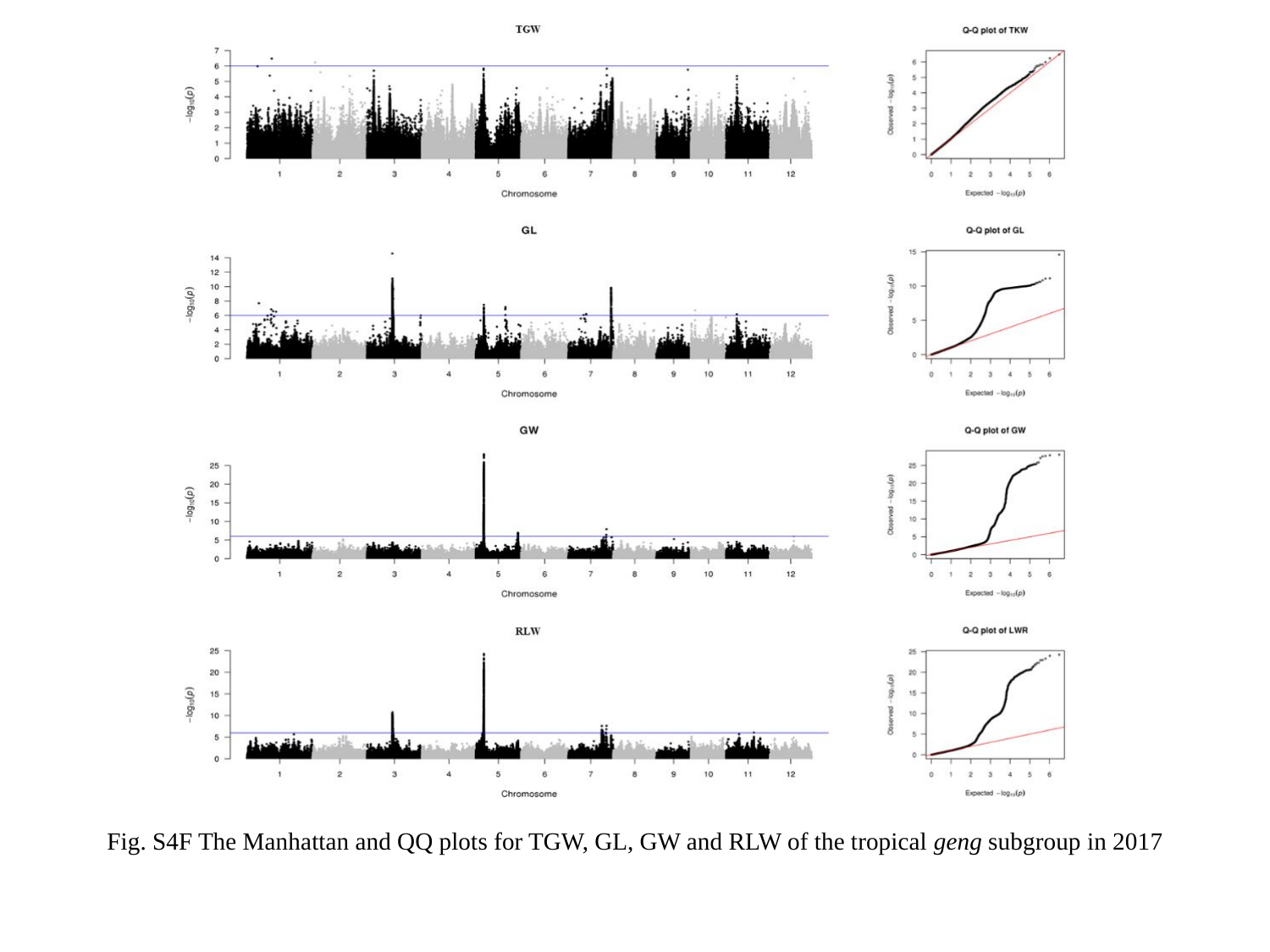

Fig. S4F The Manhattan and QQ plots for TGW, GL, GW and RLW of the tropical geng subgroup in 2017

## Slide 20
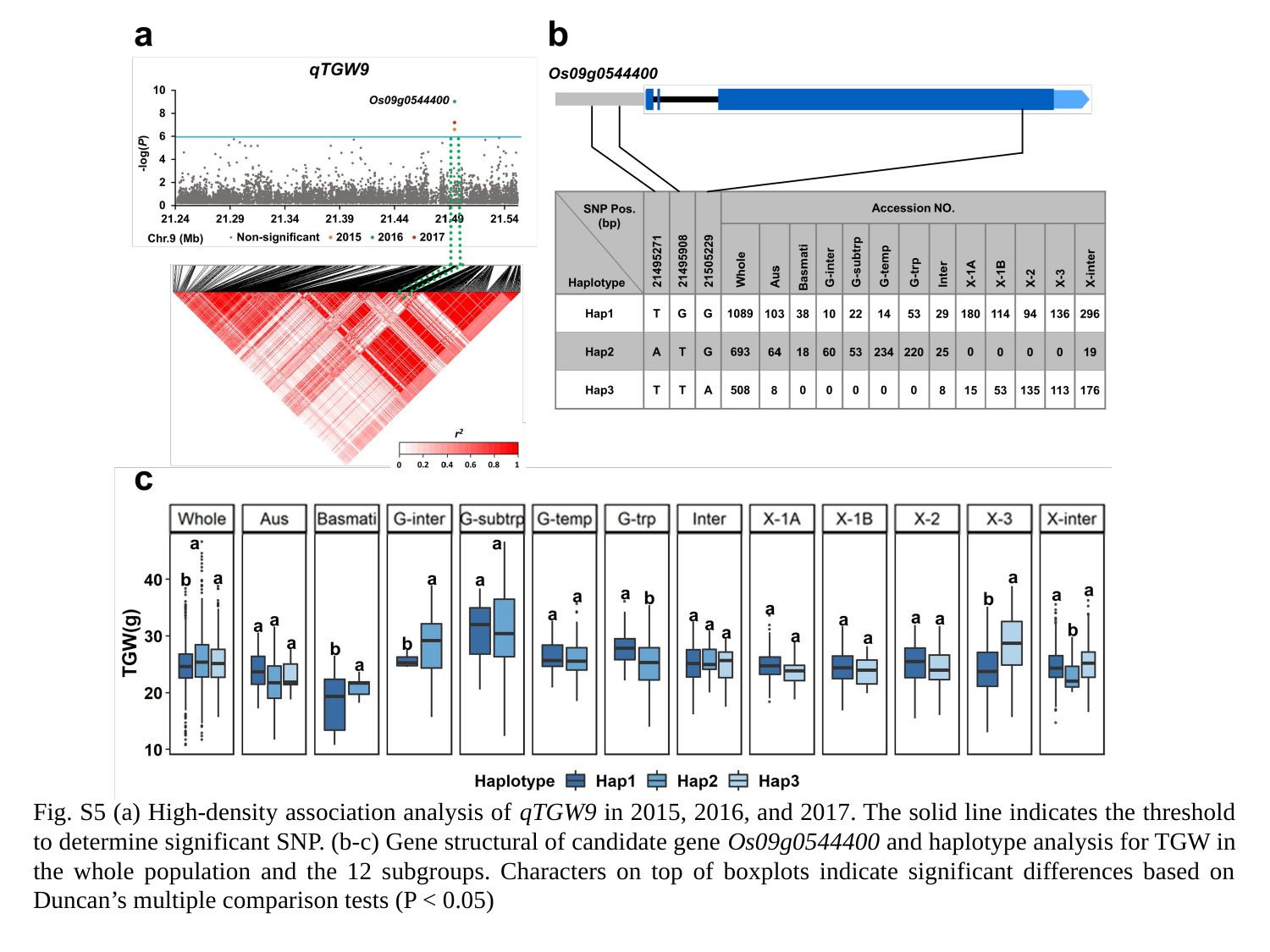

Fig. S5 (a) High-density association analysis of qTGW9 in 2015, 2016, and 2017. The solid line indicates the threshold to determine significant SNP. (b-c) Gene structural of candidate gene Os09g0544400 and haplotype analysis for TGW in the whole population and the 12 subgroups. Characters on top of boxplots indicate significant differences based on Duncan’s multiple comparison tests (P < 0.05)

## Slide 21
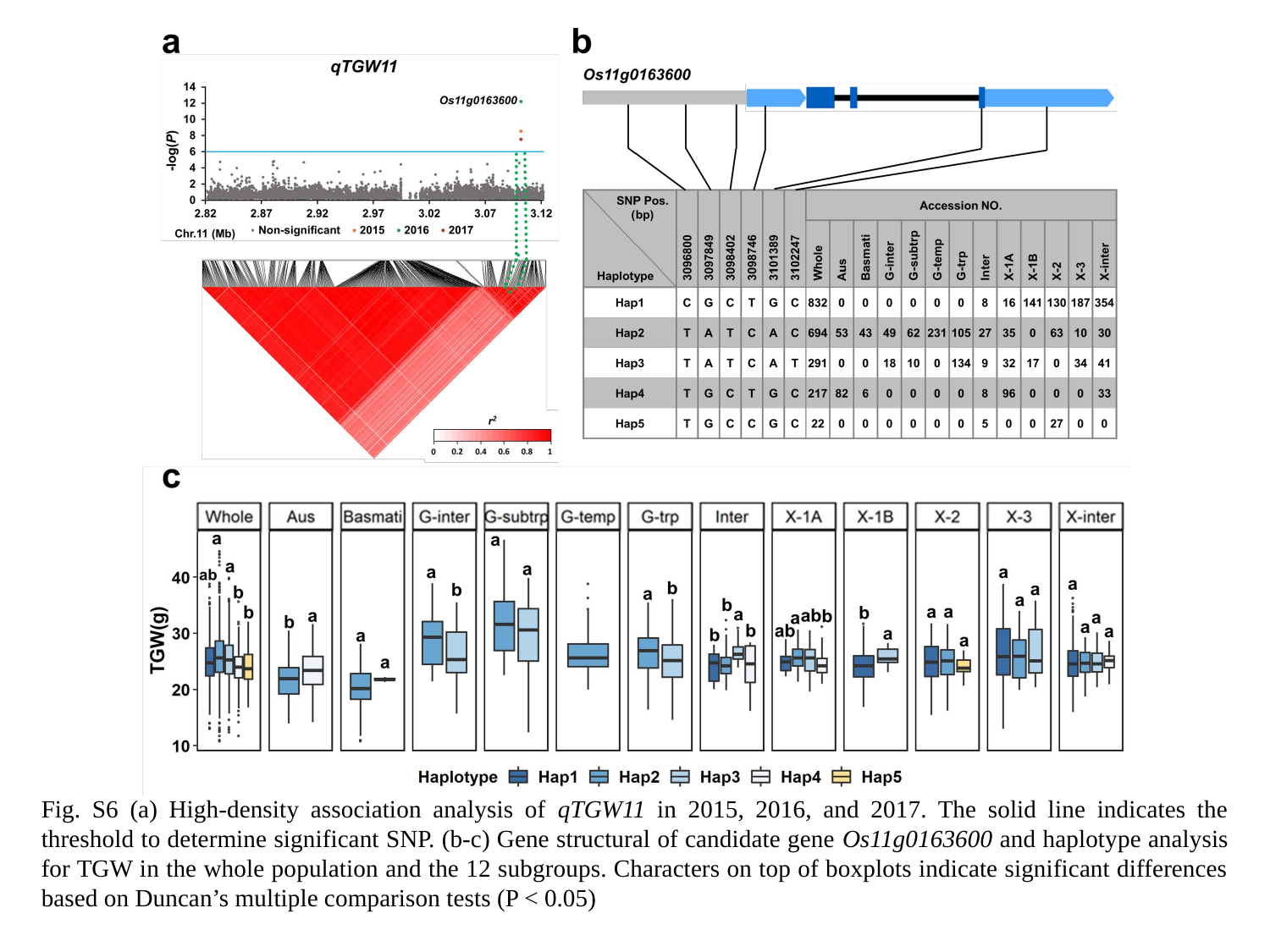

Fig. S6 (a) High-density association analysis of qTGW11 in 2015, 2016, and 2017. The solid line indicates the threshold to determine significant SNP. (b-c) Gene structural of candidate gene Os11g0163600 and haplotype analysis for TGW in the whole population and the 12 subgroups. Characters on top of boxplots indicate significant differences based on Duncan’s multiple comparison tests (P < 0.05)

## Slide 22
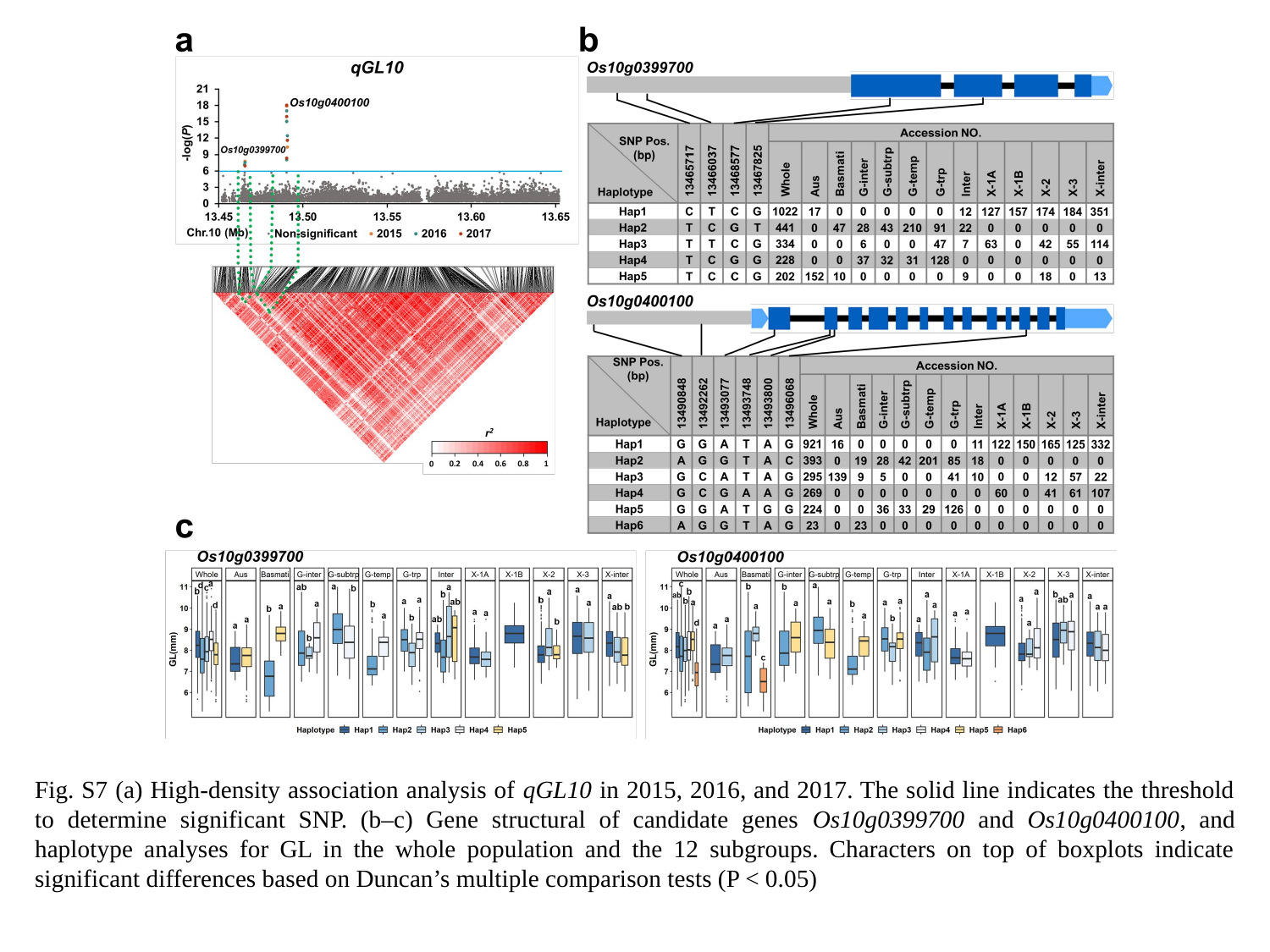

Fig. S7 (a) High-density association analysis of qGL10 in 2015, 2016, and 2017. The solid line indicates the threshold to determine significant SNP. (b‒c) Gene structural of candidate genes Os10g0399700 and Os10g0400100, and haplotype analyses for GL in the whole population and the 12 subgroups. Characters on top of boxplots indicate significant differences based on Duncan’s multiple comparison tests (P < 0.05)

## Slide 23
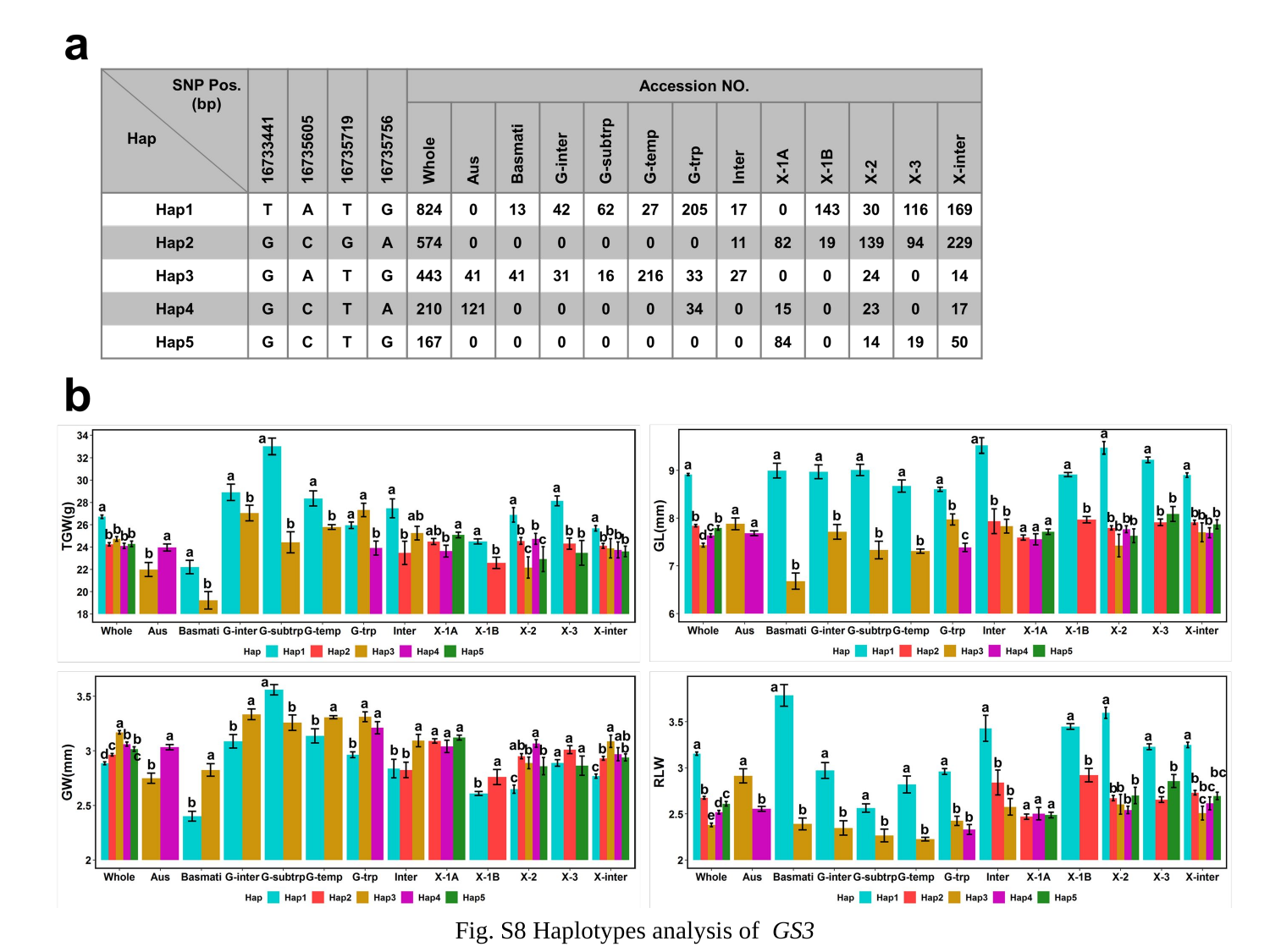

Fig. S8 Haplotypes analysis of GS3

## Slide 24
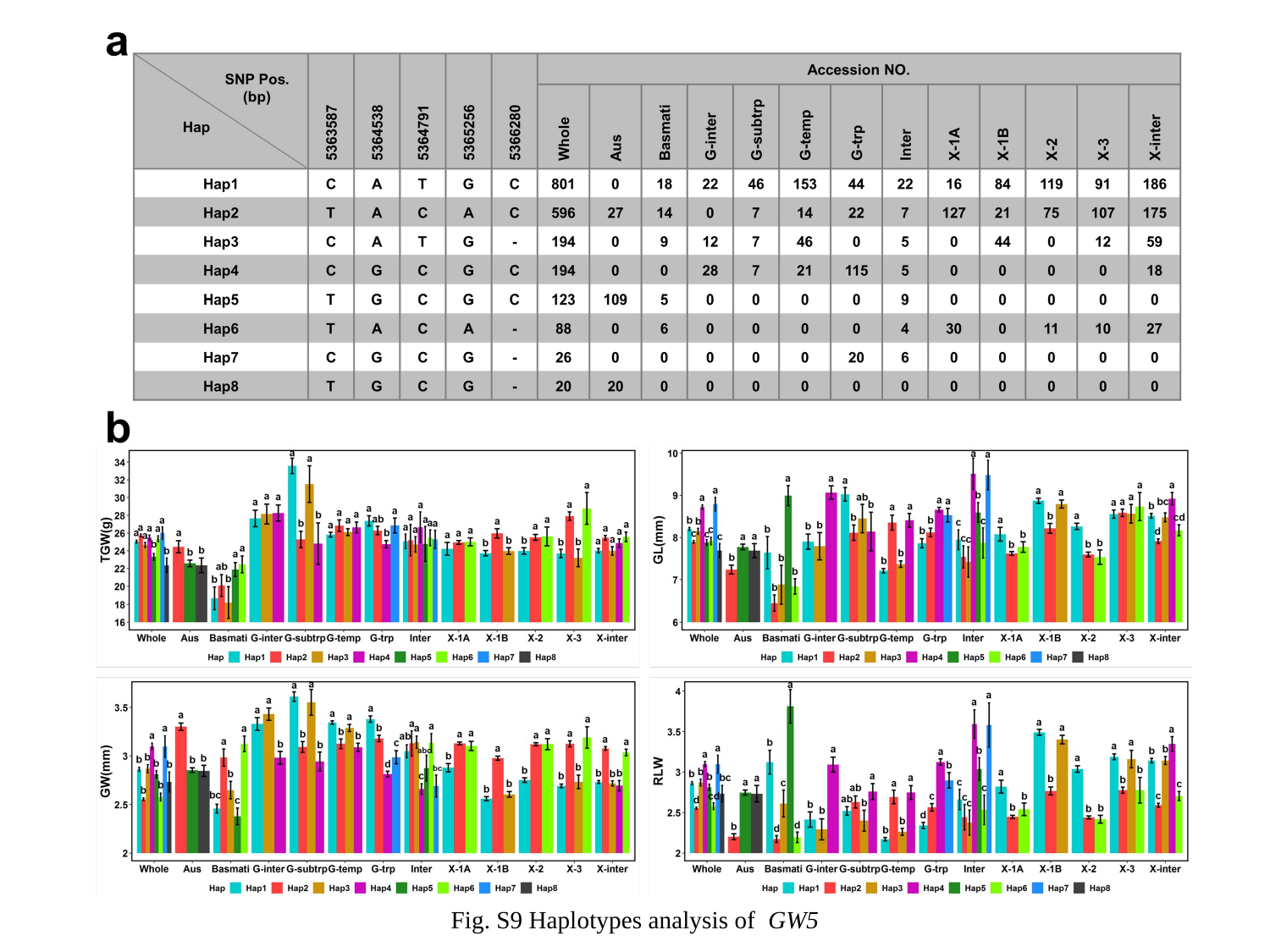

Fig. S9 Haplotypes analysis of GW5

## Slide 25
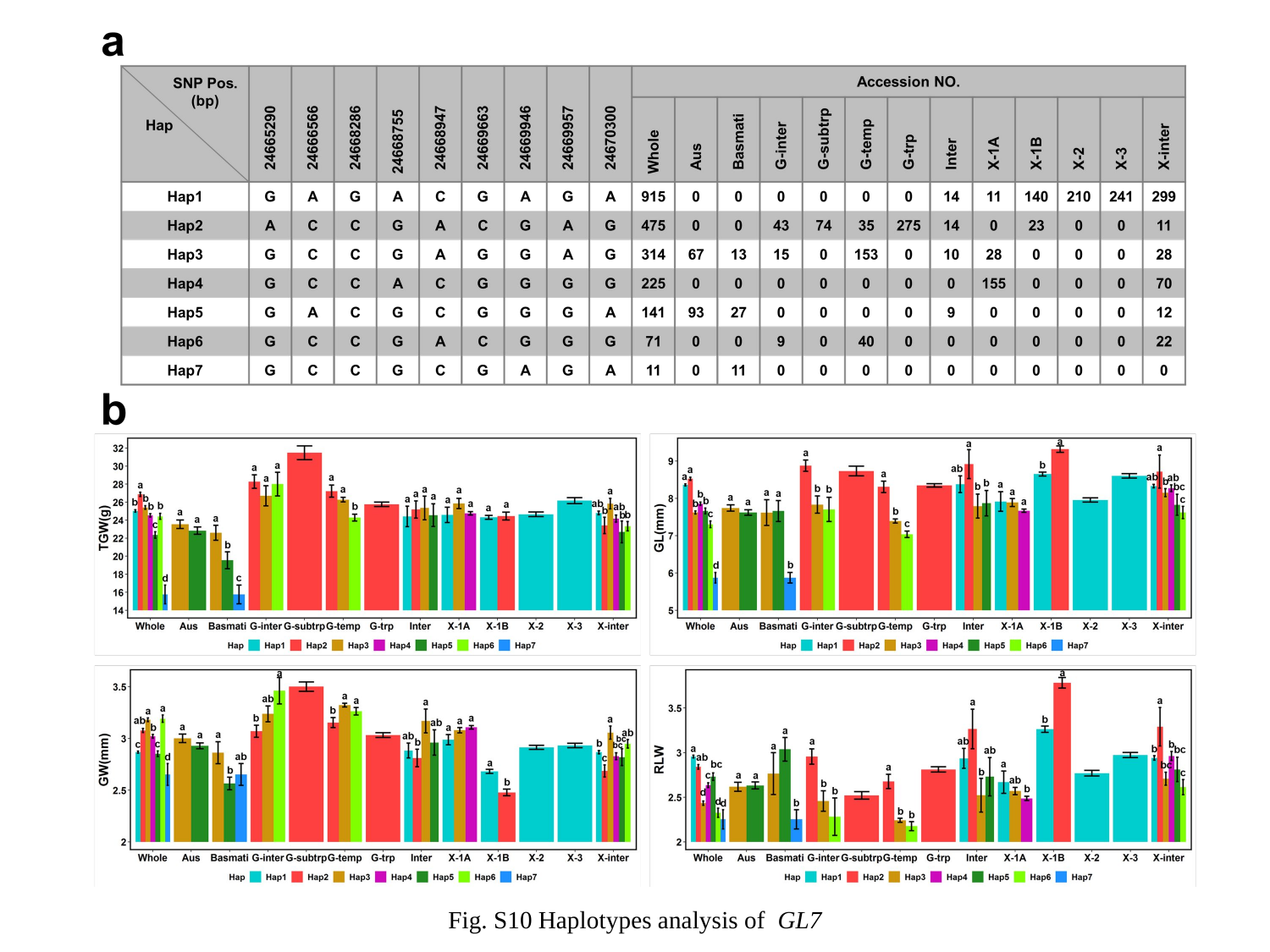

Fig. S10 Haplotypes analysis of GL7

## Slide 26
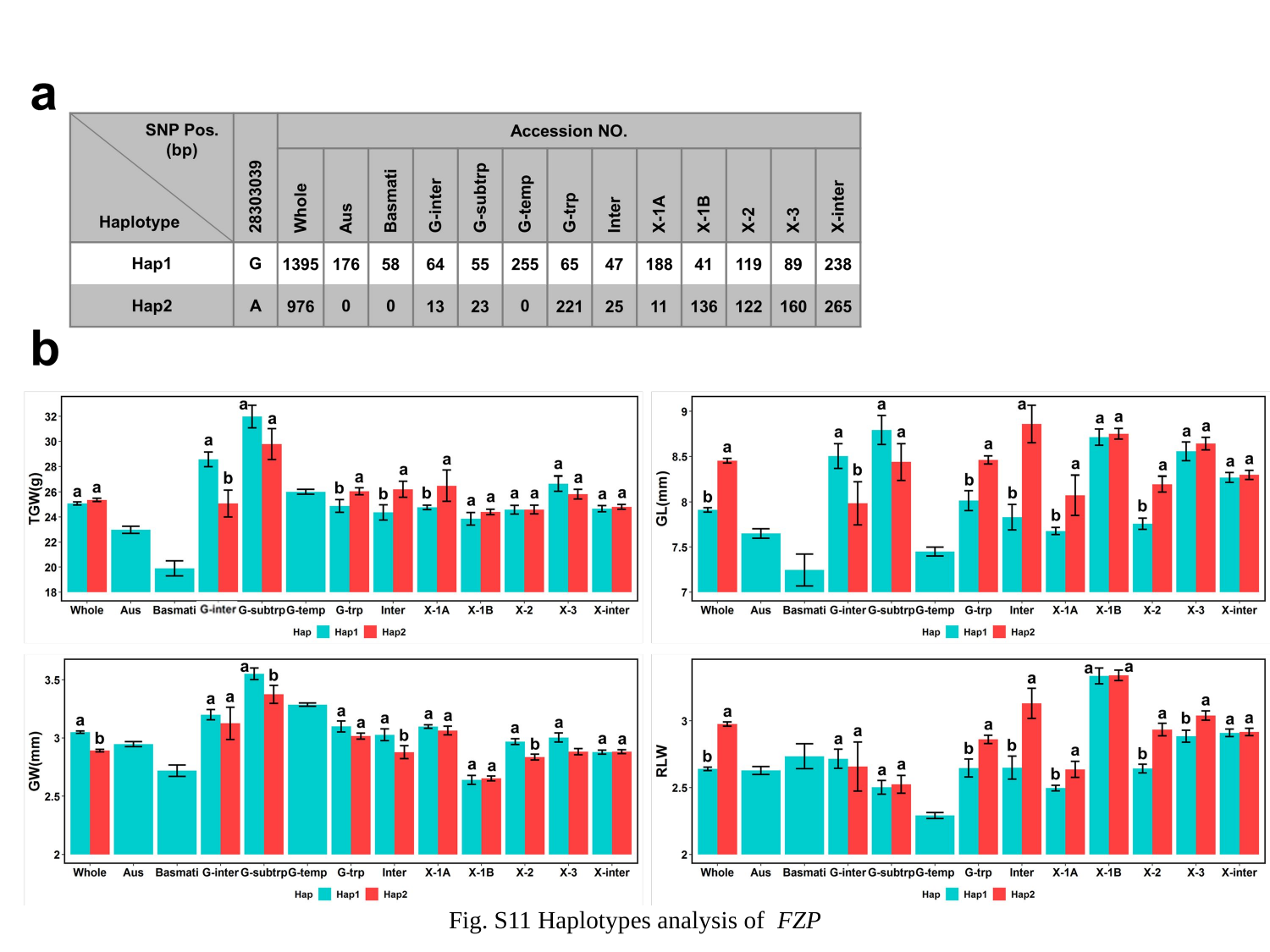

Fig. S11 Haplotypes analysis of FZP

## Slide 27
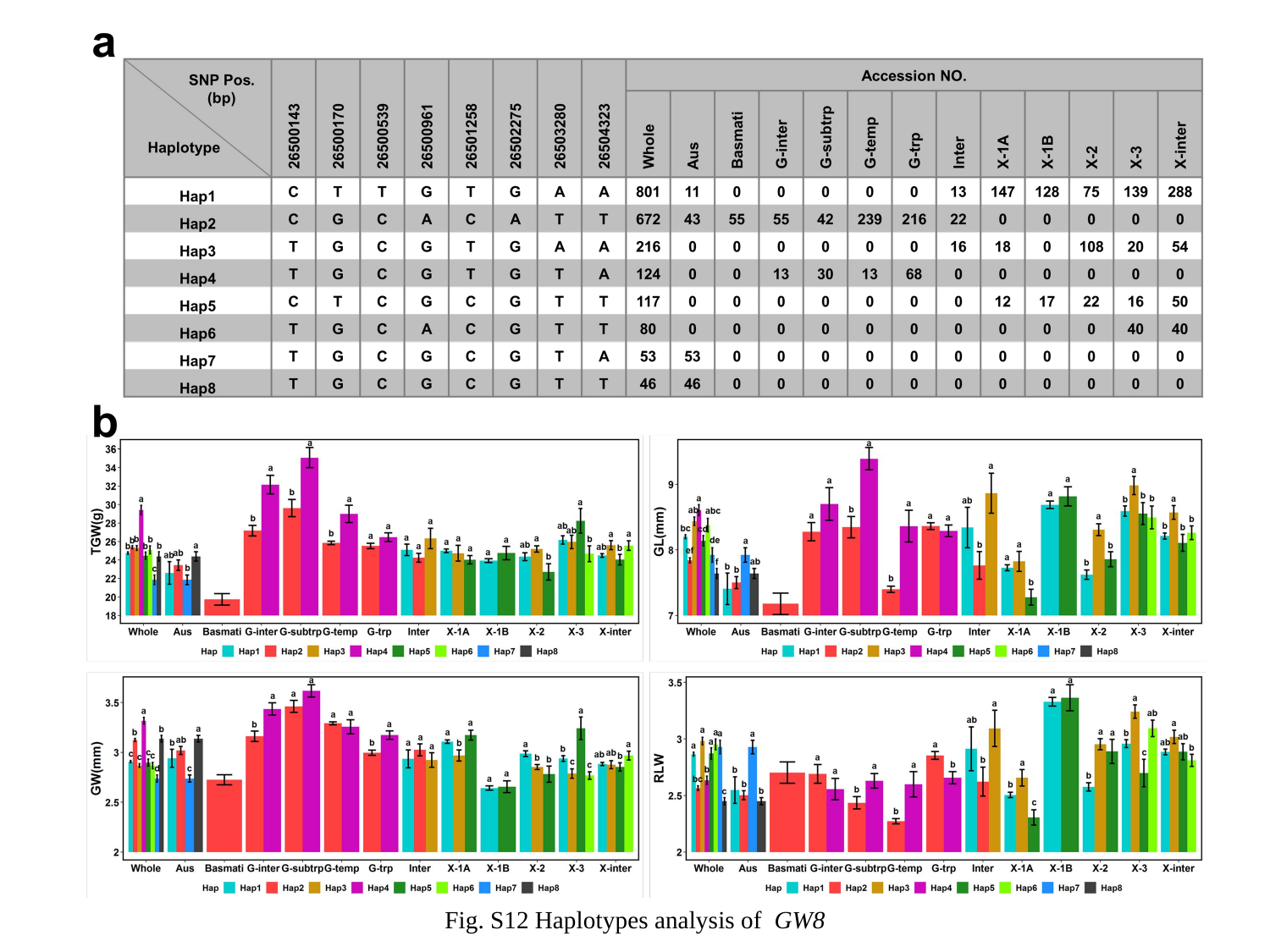

Fig. S12 Haplotypes analysis of GW8
